# Integrative single-cell analysis: dissecting CD8 + memory cell roles in LUAD and COVID-19 via eQTLs and Mendelian Randomization

**DOI:** 10.1186/s41065-023-00307-7

**Published:** 2024-01-31

**Authors:** Jintao Wu, Xiaocheng Mao, Xiaohua Liu, Junying Mao, Xianxin Yang, Xiangwu zhou, Lu Tianzhu, Yulong Ji, Zhao Li, Huijuan Xu

**Affiliations:** 1 Nanchang University Jiangxi Medical College, Nanchang, Jiangxi China; 2 Departments of Blood Transfusion, Institute of Transfusion, Jiangxi Key Laboratory of Transfusion, The First Affiliated Hospital of Nanchang University, Nanchang, Jiangxi China; 3 Key Laboratory of Jiangxi Province for Transfusion Medicine, The First Affiliated Hospital of Nanchang University, Nanchang, Jiangxi China; 4 The First People’s Hospital of Wenling, Affiliated Wenling Hospital, Wenzhou Medical University, Taizhou, Zhejiang China; 5 The Fifth Affiliated Hospital of Jinan University, Heyuan, Guangdong China; 6 The Fifth Affiliated Hospital of Shantou University, Shantou, Guangdong China; 7 Department of Thoracic Surgery, Jiangxi Cancer Hospital, Nanchang, Jiangxi Province China; 8 Department of Radiation Oncology, Jiangxi Cancer Hospital of Nanchang University, Nanchang, Jiangxi People’s Republic of China 330006; 9 NHC Key Laboratory of Personalized Diagnosis and Treatment of Nasopharyngeal Carcinoma, Jiangxi Cancer Hospital of Nanchang University), Nanchang, Jiangxi 330006 People’s Republic of China; 10 Jiangxi Key Laboratory of Translational Cancer Research, Jiangxi Cancer Hospital, Jiangxi Province, China; 11 Department of Clinical Laboratory, Hangzhou TCM Hospital Affiliated to Zhejiang Chinese Medical University, Hangzhou, China

**Keywords:** Single-cell transcriptomics, Immune function, Lung adenocarcinoma, COVID-19, Mendelian randomization

## Abstract

Lung adenocarcinoma exhibits high incidence and mortality rates, presenting a significant health concern. Concurrently, the COVID-19 pandemic has emerged as a grave global public health challenge. Existing literature suggests that T cells, pivotal components of cellular immunity, are integral to both antiviral and antitumor responses. Yet, the nuanced alterations and consequent functions of T cells across diverse disease states have not been comprehensively elucidated. We gathered transcriptomic data of peripheral blood mononuclear cells from lung adenocarcinoma patients, COVID-19 patients, and healthy controls. We followed a standardized analytical approach for quality assurance, batch effect adjustments, and preliminary data processing. We discerned distinct T cell subsets and conducted differential gene expression analysis. Potential key genes and pathways were inferred from GO and Pathway enrichment analyses. Additionally, we implemented Mendelian randomization to probe the potential links between pivotal genes and lung adenocarcinoma susceptibility. Our findings underscored a notable reduction in mature CD8 + central memory T cells in both lung adenocarcinoma and COVID-19 cohorts relative to the control group. Notably, the downregulation of specific genes, such as TRGV9, could impede the immunological efficacy of CD8 + T cells. Comprehensive multi-omics assessment highlighted genetic aberrations in genes, including TRGV9, correlating with heightened lung adenocarcinoma risk. Through rigorous single-cell transcriptomic analyses, this investigation meticulously delineated variations in T cell subsets across different pathological states and extrapolated key regulatory genes via an integrated multi-omics approach, establishing a robust groundwork for future functional inquiries. This study furnishes valuable perspectives into the etiology of multifaceted diseases and augments the progression of precision medicine.

**Supplementary Information:**

The online version contains supplementary material available at 10.1186/s41065-023-00307-7.

## Introduction

Lung Adenocarcinoma (LUAD) represents a prevalent subtype of lung cancer, and its incidence ranks among the highest globally, especially among smokers [1]. The prevalence varies across different regions, yet it constitutes a significant proportion of all lung cancer cases. The 5-year survival rate in late stages is relatively low primarily due to the typical late-stage diagnosis when the disease has already advanced; however, early detection and improved treatment methods can significantly enhance survival chances [2]. Treatment for LUAD is generally comprehensive, encompassing surgery, chemotherapy, radiation therapy, targeted therapy, and immunotherapy [3–5]. Currently, researchers are exploring biomarkers for diagnosing, prognosticating, and monitoring LUAD, with the aim of identifying superior treatment plans for patients [6, 7].

The COVID-19 pandemic, triggered by the SARS-CoV-2 infection, presents a spectrum of outcomes ranging from asymptomatic infections to life-threatening viral pneumonia and Acute Respiratory Distress Syndrome (ARDS) [8]. While host factors like age, gender, and Body Mass Index (BMI) are known to correlate with disease severity, they don't fully explain the observed inter-individual variations [9]. Despite the availability of COVID-19 vaccines, treating the disease remains imperative [10]. Nonetheless, numerous uncertainties persist regarding the genetic underpinnings of susceptibility to SARS-CoV-2 infection and the genetic determinants of COVID-19 severity. Early studies have suggested that the genes dictating an individual's blood type might impact susceptibility to SARS-CoV-2, and other immunity-related genes could also affect infection risk [11]. Polymorphisms in the ACE2 gene and the presence of TMPRSS2 (Transmembrane Serine Protease 2) are believed to increase the risk of SARS-CoV-2 infection. Moreover, research has shown that males are more susceptible to SARS-CoV-2 infection than females [12]. Several variants affecting the expression of ACE2 and TMPRSS2 receptors related to COVID-19 have been associated with susceptibility and risk factors for the disease [13]. Some genetic studies have identified potential gene variants correlated with susceptibility to SARS-CoV-2 infection and severity of COVID-19. For instance, certain gene variants might affect the binding and entry of the virus to cell surface receptors, thereby influencing infection risk and disease severity [14]. Beyond genetic factors, disease severity is also associated with age, gender, BMI, and medical history. Ethnicity too correlates with susceptibility and severity of COVID-19 [15]. By elucidating the genetic determinants of COVID-19 severity and susceptibility to SARS-CoV-2 infection, risk stratification for prioritizing immunization among high-risk individuals is feasible. Furthermore, a deeper understanding of these genes could guide personalized treatment approaches [14]. These studies and findings illustrate an evolving understanding of COVID-19, yet numerous unknown aspects require further exploration. With the accumulation of more research and data, a better grasp of the genetic basis of SARS-CoV-2 infection, and how genetic information can be utilized to assess and mitigate COVID-19 risk is anticipated.

Single-cell technology, a revolutionary research method, elucidates cellular heterogeneity and functional diversity by analyzing various biological attributes of individual cells. Through single-cell technology, researchers can delve into cell states, activities, and interactions among cells, enriching multifaceted studies in the life sciences domain. The continuous advancement of single-cell technology, especially in multiplex analysis, high throughput, high resolution, and accuracy, has enabled a comprehensive depiction of a cell's genetic landscape [16]. Recent progress includes single-cell epigenomics, single-cell genomics for lineage tracing, spatially resolved single-cell transcriptomics, and single-cell omics sequencing technologies based on third-generation sequencing platforms [17]. Due to rapid technological developments, encompassing improvements in throughput, accuracy, automation, and commercialization, single-cell RNA sequencing (scRNA-seq) has been extensively utilized to address pivotal biological and medical questions [17]. Single-cell multi-omics technology has impacted cell lineage tracing, the creation of tissue and cell-specific atlases, tumor immunology, and cancer genetics, alongside fundamental and translational research in cellular spatial information mapping [16]. These technologies have been deployed in research concerning tumors, microbiology, neurology, reproduction, immunology, digestive, and urinary systems, unveiling the crucial role of single-cell sequencing in both fundamental and clinical research [18]. Single-cell technology facilitates the generation of cell and tissue atlases, exploration of complex disease biology, thus offering a potent tool to solve various problems in the life sciences domain [16]. Recently, an increasing number of researchers are employing single-cell sequencing technologies to investigate cell-level specific mechanisms, cell phenotypes, and cell-specific gene expression, aiming for a better understanding of cellular biological characteristics and functions. The ongoing progress and widespread application of single-cell technology are propelling multifaceted research in life sciences, providing robust support to address key questions in biology and medicine. As the technology continues to evolve, broader applications of single-cell technology are envisioned, and its potential to significantly contribute to future scientific research is anticipated.

Mendelian Randomization (MR) epitomizes a robust statistical methodology for exploring causal associations, particularly playing a pivotal role in drug target research. This method leverages common genetic variants as an unconfounded and unbiased "natural randomized trial" to address causality issues [19]. MR has been extensively applied to various disease research endeavors. For instance, in COVID-19 research, investigators can employ MR to probe the association between specific gene variants and viral infection, as well as how these variants influence the course and severity of the disease post-infection [20]. In Parkinson's disease research, MR analysis could elucidate whether a causal link exists between specific gene variants and the risk of developing Parkinson’s disease [21]. Mendelian Randomization, a potent tool for causal inference, offers opportunities to delve into the relationship between diseases and drug treatment efficacy. By thoroughly comprehending the principles and applications of this method, a better understanding of disease pathogenesis and potential treatment strategies is achievable.

**Fig. 1:**
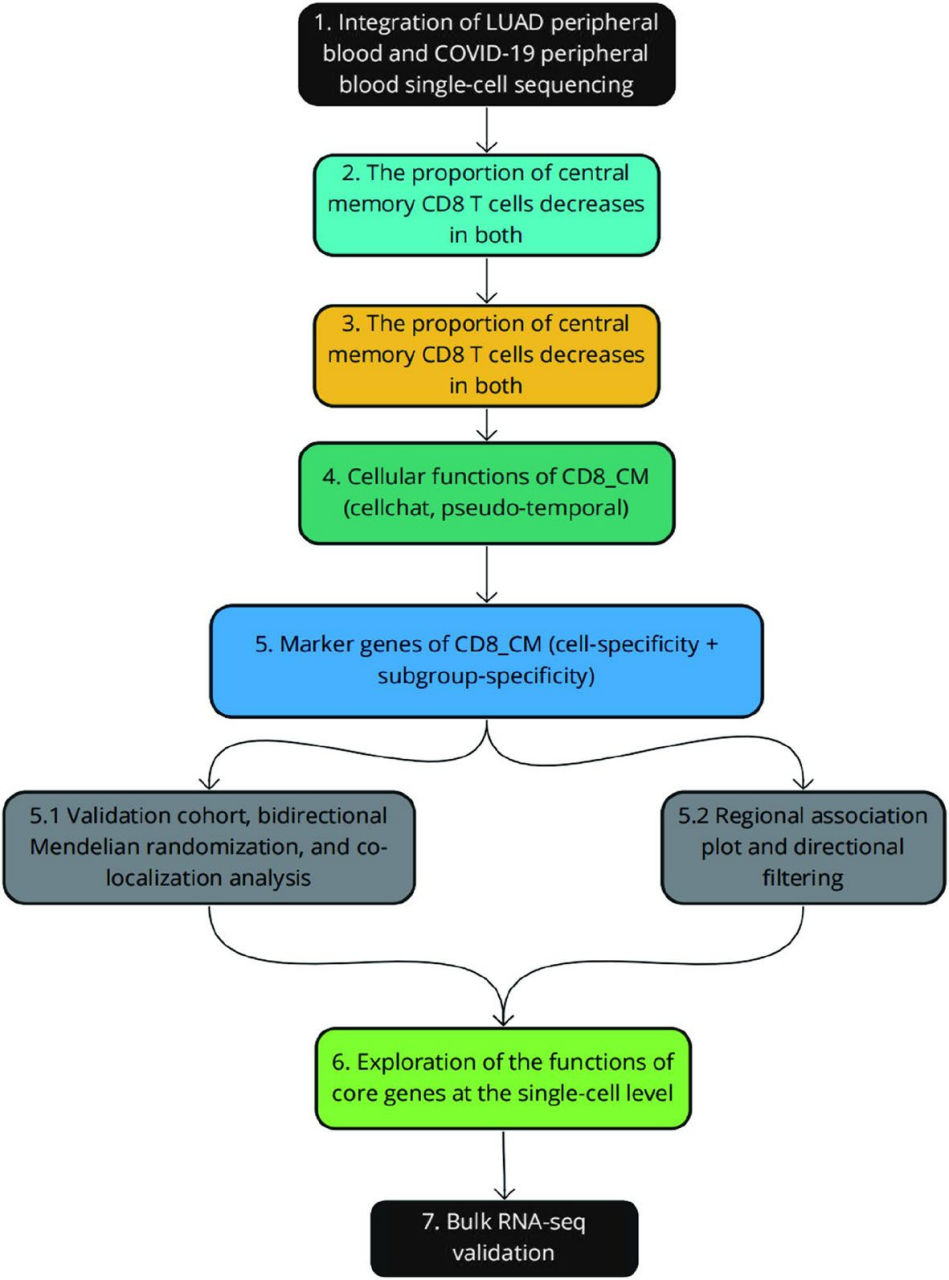
The workflow of the present study

In this study, we aim to identify potential targets between COVID-19 and lung cancer, conducting MR analysis by integrating eQTL discovered in blood with two independent LUAD genome-wide association study (GWAS) datasets. The association between TRGV9 and lung cancer was examined. Our study represents a secondary analysis of publicly available data. According to the original GWAS protocols, all participants provided informed consent, and all ethical approvals for the GWAS were obtained by the original GWAS authors. The workflow of this study is illustrated in Fig. 1.

## Methods

### Data collection

We downloaded STAR-counts data and clinical information from both 54 normal and 503 LUAD samples from the TCGA dataset repository (https://portal.gdc.cancer.gov). From this dataset, we extracted TPM-formatted data, subsequently undergoing normalization processing via log2 (TPM + 1). We retained samples possessing RNAseq data and clinical information for further analyses. The samples were utilized for subsequent analyses. The log-rank test was employed to assess the survival differences between the aforementioned two or more groups using Kaplan–Meier survival analysis. Datasets GSE171555 and GSE162498 were retrieved from the GEO database (https://www.ncbi.nlm.nih.gov/). We selected samples from three COVID-19 infected patients (GSM5227108, GSM5227109, GSM5227110), three healthy individuals (GSM5227117, GSM5227130, GSM5227134), and three peripheral blood samples from patients with lung adenocarcinoma (GSM4952957, GSM4952958, GSM4952959) for 10X single-cell sequencing. The FastQC tool assessed sequencing data quality, and Cell Ranger was employed for read mapping and count matrix generation. We also accessed the GSE43458 dataset as an external cohort for bulk-RNA analysis and risk model validation. To ascertain the potential causal link between differentially expressed genes and lung adenocarcinoma, we executed bidirectional Mendelian Randomization (MR) on two distinct datasets. Initially, we treated differentially expressed genes as risk factors with lung adenocarcinoma as the outcome and subsequently reversed this approach. A two-step MR method then evaluated the regulatory roles of pivotal genes in lung adenocarcinoma. The SNPs utilized as genetic instruments were derived from a comprehensive European GWAS (https://gwas.mrcieu.ac.uk/) [22, 23].

### Single-cell data processing and analysis

In our study, we extracted data from various samples including normal, COVID-19 infected, and tumor tissues, and generated a Seurat object for each. These objects were then amalgamated into a single comprehensive Seurat object, facilitating unified analysis. To ensure data integrity, we assessed the proportion of mitochondrial and erythrocyte genes in each cell, applying quality control criteria to exclude cells of suboptimal quality. We established a gene count range of 200 to 4000 and a mitochondrial gene proportion threshold of less than 10% for quality control. Subsequently, data normalization was performed to mitigate inter-sample variability. During dimensionality reduction and clustering, we initially selected 2000 genes with the highest variability for principal component analysis (PCA). To further minimize batch effects, we employed the Harmony [24] algorithm and visualized the data in two dimensions using the UMAP technique, which revealed various cell subpopulations in an unsupervised manner. Cell subpopulation annotation leveraged the Cell Marker database, with representative marker genes displayed via VlnPlot and FeaturePlot. Manual cell type labels were assigned to each subpopulation and annotations were preserved. In addition to manual annotation, we explored automated annotation with the SingleR package, presenting the results through DimPlot. Following satisfactory annotation, we proceeded with differential gene expression analysis, adopting a 1.5-fold change and an adjusted *p*-value of less than 0.05 as significance criteria, showcased via VolcanoPlot. Differential genes were functionally annotated and their expression across cell subpopulations was depicted in a heatmap. For data encompassing temporal sequences or developmental processes, we utilized the Monocle method for cell trajectory reconstruction and the cellchat package to examine intercellular communication and regulatory dynamics.

### CD8_CM Key marker gene eQTL and LUAD's Mendelian randomization analysis

Gene expression data preprocessing involved normalization, batch effect mitigation, and missing value handling. Key marker genes of CD8_CM, in comparison with other T cells and cells, were identified. An eQTL analysis for the CD8_CM marker gene followed. Gene symbols converted to ENSEMBL IDs ensured data uniformity. Low-quality SNPs underwent removal, and genotype fitting occurred. A stringent eQTL *P*-value threshold, such as 5 × 10^-8, was set. From the 'finn-b-C3_NSCLC_ADENO_EXALLC' GWAS dataset, SNPs related to the key marker gene were extracted as MR analysis instrumental variables. Following the R^2 and F-statistic calculations for each SNP, only high-quality SNPs remained. Outcome data corresponding to these instrumental variables facilitated Mendelian Randomization analysis using the TwoSampleMR package.

### Validation set MR analysis, bidirectional Mendelian MR and colocalization analysis

The validation set underwent initial MR analysis. By combining outcome and instrumental variable data, a harmonized dataset ('harmonised_dat') emerged. The MR analysis utilized a 'mr_modified' function, estimating a gene's causal impact on the target disease and computing the Phenotypic Variance Explained (PVE). Visualization tools like the 'forest' function presented the MR results. Subsequently, a Bidirectional Mendelian Randomization analysis was conducted. Instrumental variable data linked to the reverse Mendelian disease ("ieu-a-985") were extracted and merged with the corresponding outcome data to produce a 'harmonized LUAD gene.' The vcfR package processed the gene expression's eQTL data. Post data organization and merging, a colocalization analysis was conducted, employing the 'coloc.abf' function to evaluate Bayes factors under various colocalization hypotheses. The results were then interpreted in light of the Bayes factors' magnitude and distribution.

### Regional association plot, phenoscanner analysis and directional filtering

Initially, we constructed the regional association plot. By reading the genotype data eqtl-a-ENSG00000211695.vcf (in VCF format) and related association data, we extracted eQTL information relevant to the target gene. Based on this, eQTLs located within a specified region were selected and organized into a format suitable for drawing the regional association plot. The drawing process utilized the locuscomparer package to visualize the association information of eQTL and GWAS, providing an intuitive graphical presentation for subsequent analysis. Next, PhenoScanner analysis was conducted. By loading existing GWAS summary statistics, the custom mr_phenoscanner function was used to query the association information of each SNP in directories such as GWAS, eQTL, and pQTL. This process employed the PhenoScanner tool to query different directories based on SNP associations and to organize the results. Finally, through Steiger filtering, for each trait and PMID combination, the most significant SNP was screened and summarized into a comprehensive result table. Lastly, a directional filtering analysis was conducted. The steiger_filtering function was applied to the SNPs in harmonised_dat to perform the Steiger test, assessing the R2 difference between SNP with exposure and SNP with outcome. This aids in verifying the position of SNP in the causal chain. The directionality_test function was also used to test the directional relationship between the SNP, exposure, and outcome.

### Exploring the function of exposure factors at the single-cell level

First, through single-cell RNA expression analysis, we explored the expression of the target gene at the single-cell level and used visualization tools such as DotPlot and FeaturePlot to display the expression patterns of key genes. Next, trajectory analysis was conducted. Using the slingshot tool, we constructed cell developmental trajectories and visualized them with UMAP. In addition, key genes driving the trajectory were analyzed. Functions like find_switch_logistic_fastglm were employed to identify switch genes that might play an essential role in the trajectory development. The plot_timeline_ggplot was used to depict gene expression patterns over pseudotime. Subsequently, cell communication analysis was executed. Using the CellChat tool, communication between different cell clusters was analyzed, including identifying ligand-receptor pairs, projecting onto protein interaction networks, and calculating communication probabilities. Functions like netVisual_circle and netVisual_bubble visually displayed the communication network structure between different cell clusters. In metabolic analysis, the scMetabolism tool was used to assess the metabolic activity of macrophages. We presented the activity differences of cells from different gene groups in specific metabolic pathways using the metabolism pathway's DotPlot. Finally, differential gene analysis was conducted. Using Seurat's FindAllMarkers function, differentially expressed genes in the CD8_CM cell subgroup were identified. The pheatmap was utilized to draw the heatmap of the expression matrix, displaying the gene expression patterns across samples. Differential gene analysis was also conducted at the bulk data level by reading external datasets.

### Trajectory inference

We employed the monocle3 R package [25, 26] to infer the differentiation trajectories of T cells. Initially, the data underwent preprocessing steps including quality control, normalization, and dimensionality reduction. Subsequently, following the guidelines provided by the official documentation, we configured the parameters before conducting cell state inference and transcriptomic trajectory analysis.

### Cell culture

A549, PC-9, and H1299 cell lines obtained from the Cell Bank of the Chinese Academy of Sciences. The cells were cultured at 37 °C with a high-glucose medium containing 10% fetal bovine serum and 5% CO2.

### Western blotting

Using RIPA buffer supplemented with phenylmethylsulfonyl fluoride (PMSF), protease inhibitors (PI), and phosphatase inhibitors (PPI). The total protein concentration of the supernatants was quantified using the Pierce BCA Protein Assay Kit according to the manufacturer's instructions (Thermo Fisher Scientific, USA). The proteins were then separated by SDS-PAGE and transferred onto PVDF membranes (Millipore, Billerica, MA, USA) for subsequent analysis. Membranes were blocked with 5% non-fat milk in Tris-buffered saline containing 0.1% Tween-20 (TBST) for two hours at room temperature, followed by overnight incubation at 4 °C with the primary antibody TRGV9(TRGC1) (abcam, USA, ab192031). After washing thrice with TBST, the membranes were incubated with horseradish peroxidase-conjugated secondary antibody. Chemiluminescent detection was employed to visualize and capture the immunoreactive bands.

### Data analysis

All data analyses were performed based on R 4.1.3, with *p* < 0.05 considered statistically significant.

## Results

### Single-cell transcriptomic analysis of lung adenocarcinoma, COVID-19, and normal groups

**Fig. 2:**
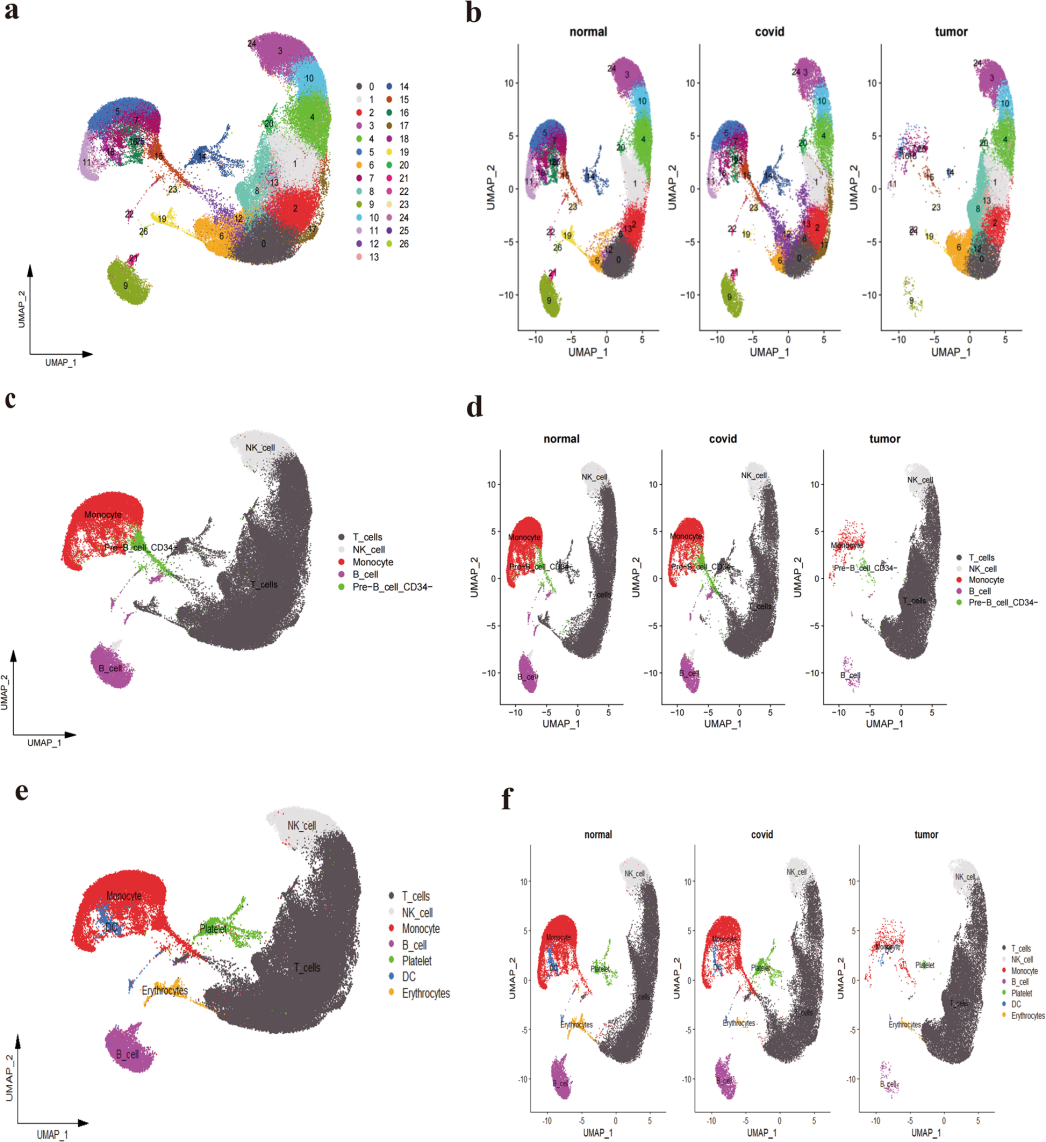
Single-cell transcriptomics landscape of various samples. **a**, **b** Depict the single-cell transcriptomic profiles of different samples, showcasing the diversity and distribution of cell clusters. **c**, **d** Using the Single R package, we annotated the 26 clusters. The results of this automated classification are depicted, showing various cell populations. **e**, **f** Post manual annotation, seven distinct cell populations were identified: T_cells, NK_cell, Monocyte, B_cell, platelet, DC, and Erythrocytes

In this study, we selected three COVID-19 patient samples, three healthy controls, and three sets of peripheral blood from lung adenocarcinoma patients for 10X single-cell RNA-seq analysis from datasets GSE171555 and GSE162498. Initial screening was performed to eliminate low-quality data (Supplementary Fig. 1a), retaining 56,851 cells for subsequent analysis. Data normalization was displayed (Supplementary Fig. 1b), and to mitigate batch effects among different samples, we integrated and standardized the samples using the Harmony method, followed by normalization, principal component analysis (PCA) dimensionality reduction, and clustering (Supplementary Fig. 1c). Visualization of each cluster was accomplished using the UMAP technique based on the first 15 principal components (Supplementary Fig. 1d). Specific marker genes were employed to identify and annotate different cell subpopulations within the single-cell RNA sequencing data. Using the Seurat package's VlnPlot function, we generated violin plots of marker gene expression for various cell types, including B cells, natural killer (NK) cells, T cells, monocytes, dendritic cells (DC), megakaryocytes/platelets, and erythrocytes, demonstrating their distribution across cell subpopulations (Supplementary Fig. 2a). Furthermore, the FeaturePlot function was used to generate feature distribution maps of these marker genes, visualizing their spatial distribution within cells (Supplementary Fig. 2b). We confirmed the number of cell subpopulations defined by unsupervised clustering algorithms and annotated them using the RenameIdents function, aligning each cluster with its corresponding cell type. Cell distributions were visualized using the UMAP algorithm and the DimPlot function (Fig. 2a) and segmented according to tissue type (Fig. 2b). Subsequently, we renamed 26 clusters using the Single R package and visualized cell distributions with UMAP reduction and DimPlot (Fig. 2c), segmenting by tissue type (Fig. 2d). To ensure the reliability of annotations, manual annotation was also performed, visualizing cell distributions with UMAP and DimPlot (Fig. 2e) and segmenting by tissue type (Fig. 2f) to enhance the understanding of cell type distributions across different tissues.

### GO, KEGG, and WikiPathway enrichment analysis

**Fig. 3:**
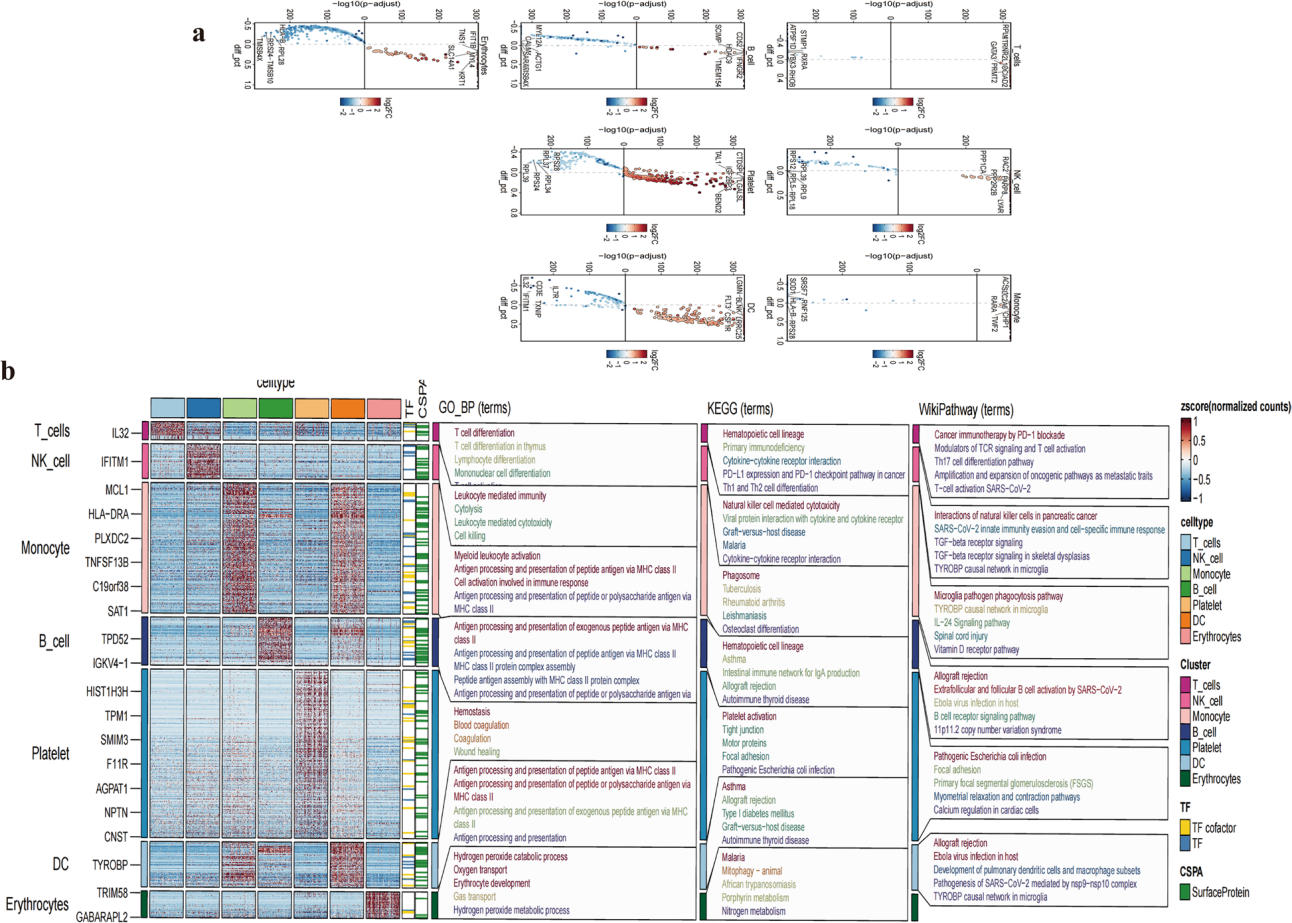
Functional analysis of differential genes (**a**). Display of the top five highly and lowly expressed genes in the 7 cell groups in the volcano plot (**b**). GO, KEGG and WikiPathway enrichment analysis

Based on the 7 cell types identified, genes with fold changes ≥ 1.5 or ≤ -1.5 were considered differentially expressed. We visualized the top 5 overexpressed and underexpressed genes in T_cells, NK_cell, Monocyte, B_cell, platelet, DC, and Erythrocytes (Fig. 3a). To decipher the function of differentially expressed genes in various cell clusters, we referenced databases like TF (Transcription Factors), CSPA (Cell Surface Protein Atlas), GO_BP (Gene Ontology Biological Processes), KEGG (Kyoto Encyclopedia of Genes and Genomes), and WikiPathway. Our results offer a preliminary understanding of the function of differentially expressed genes in each cell type (Fig. 3b).

### Single-cell transcriptomic analysis of T cells

**Fig. 4:**
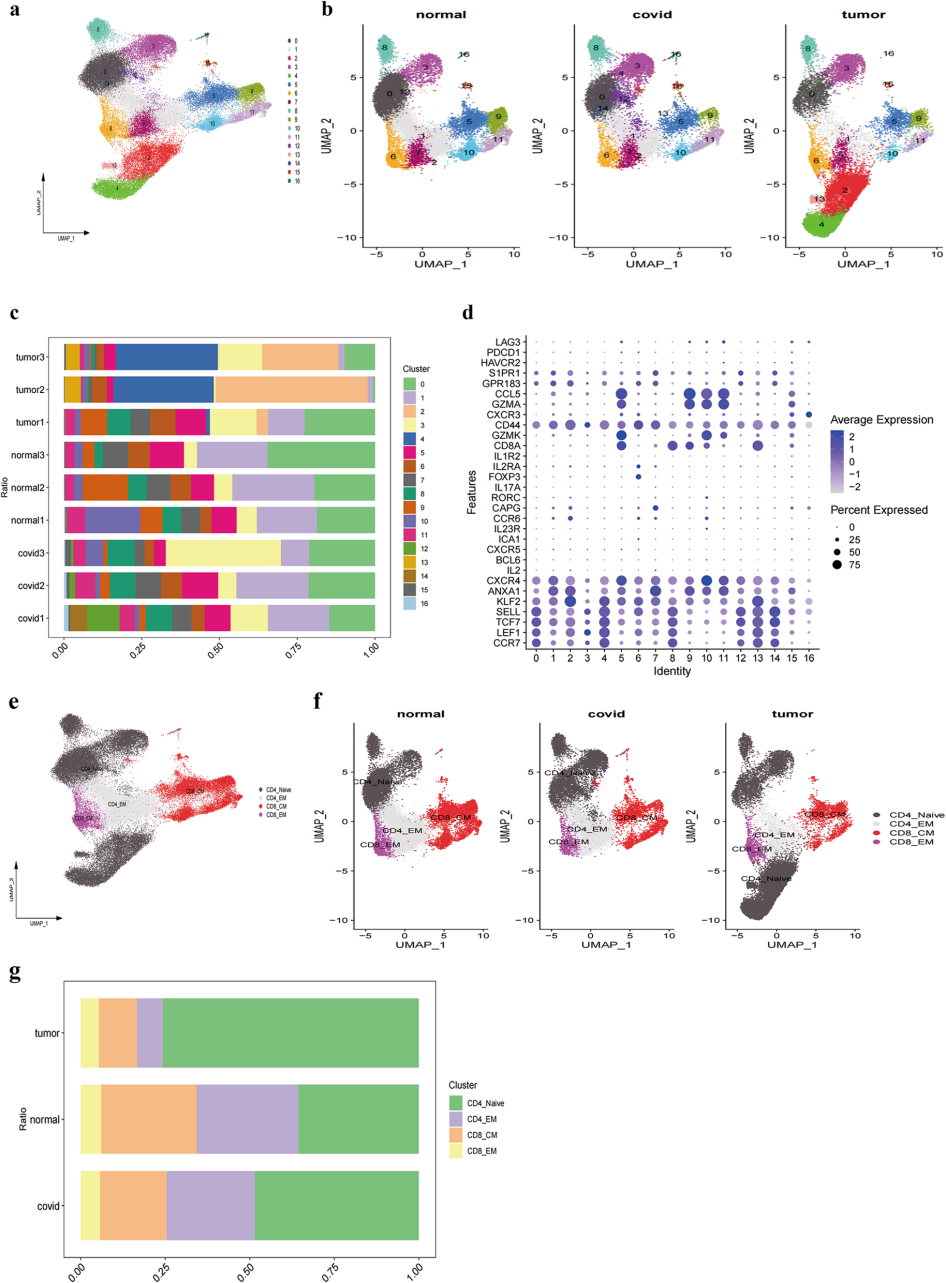
In-depth Analysis and Visualization of T-cell Subsets. **a**, **b** Featureplot showcasing the distribution of T-cells across 17 clusters. **c** Displays the proportion of each cell subset in different samples. **d** Dot Plot illustrating the expression of characteristic genes across various cell subsets. **e**,** f** Manually annotated T-cell subgroups including CD4_Naïve, CD4_EM, CD8 CM, and CD8_EM. **g** Depicts the distribution of T-cell subgroups in different samples

T cells play multifaceted roles in both cancer and COVID-19, encompassing direct cytotoxicity against infected or aberrant cells, assisting the activity of other immune cells, and maintaining immunological balance. A thorough understanding of T cell functions and interactions is crucial for the development of effective therapeutic and vaccine strategies in both contexts. Initially, T cell subpopulations were extracted from comprehensive single-cell RNA sequencing data, followed by preprocessing, dimension reduction, clustering, and visualization analyses. The distribution of 17 T cell clusters was visualized using UMAP and DimPlot (Fig. 4a), segmented by tissue type (Fig. 4b). Cell proportion charts (Fig. 4c) and Dot Plots (Fig. 4d) illustrated the distribution and expression of characteristic genes within different T cell subpopulations, providing a visual analytical foundation for a deeper understanding of their functions and characteristics. We visualized the distribution of four T cell subpopulations with UMAP and DimPlot (Fig. 4e), also segmented by tissue type (Fig. 4f). Figure 4G shows the cell proportions across different samples, where we noted a higher proportion of CD8_CM in the normal group compared to the COVID and tumor groups, and a higher proportion of CD4_Naïve cells in the COVID and tumor groups relative to the normal group.

### Enrichment analysis of four T_cell subgroups based on GO, KEGG and WikiPathway

**Fig. 5:**
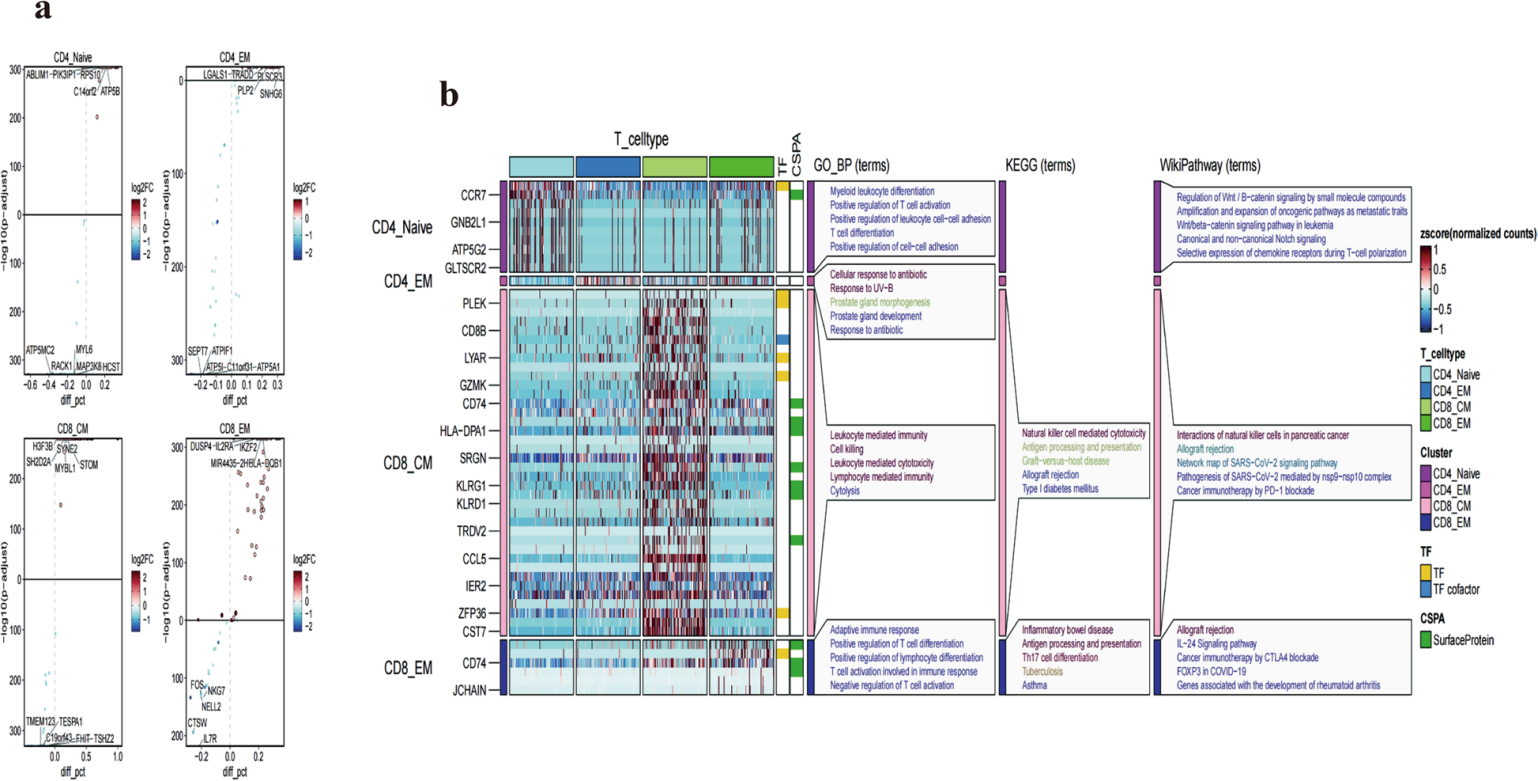
GO, KEGG and WikiPathway enrichment analysis based on 4 subgroups of T_cells (**a**). Display of the top five highly and lowly expressed genes in the 4 cell groups in the volcano plot (**b**). GO, KEGG and WikiPathway enrichment analysis

Based on the identified four cell types, differential gene expression was determined using the findallmarker function, with genes exhibiting a fold change of ≥ 1.5 or ≤ -1.5 considered significant. We visualized the top five upregulated and downregulated genes in the CD4_Naïve, CD4_EM, CD8_CM, and CD8_EM subgroups (Fig. 5a). GO_BP analysis indicated the CD4_EM group's differential genes primarily enriched in pathways related to cellular response to antibiotics and UV-B radiation. For the CD8_CM group, these genes were chiefly associated with leukocyte-mediated immunity, cell killing, and lymphocyte-mediated cytotoxicity pathways. KEGG analysis demonstrated that the CD8_CM subgroup's genes were enriched in natural killer cell-mediated cytotoxicity, whereas the CD8_EM subgroup's genes were linked to inflammatory bowel disease, antigen processing and presentation, and Th17 cell differentiation pathways. WikiPathway analysis showed enrichment in interactions of natural killer cells in pancreatic cancer for the CD8_CM group, and allograft rejection for the CD8_EM group (Fig. 5b). Collectively, these findings offer insights into the distinct functional roles and biological features of various T cell subgroups, enhancing our understanding of their involvement in immune responses and disease processes.

### Trajectory analysis and cellular communication of primary T cell types

**Fig. 6:**
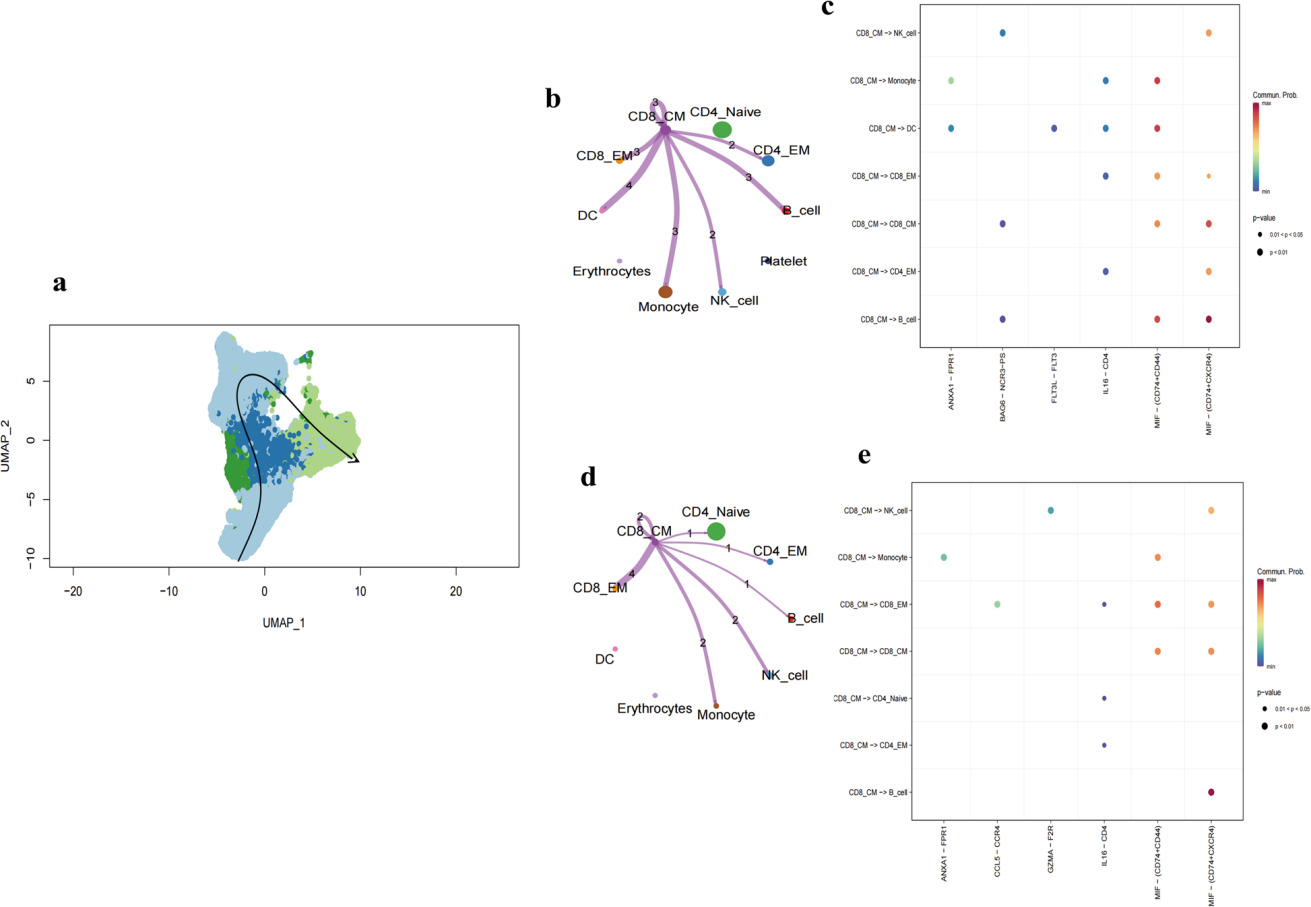
Cellular Differentiation and Communication Analysis in COVID-19 and LUAD. **a** Cellular differentiation trajectory plot using UMAP, showcasing the progression from CD4_Naïve to CD8_CM, implying that CD8_CM represents terminally differentiated CD8_T cells. **b**, **d** Cellular communication networks constructed for CD8_CM and other cells in both COVID-19 and LUAD samples. The networks highlight potential interactions and communication patterns between CD8_CM and other cell types. **c**, **e** Differential enrichment signaling pathway analysis between CD8_CM and other cells

T cell development originates in the bone marrow, where lymphocyte precursor cells undergo a series of differentiation steps, inclusive of T cell precursors. This differentiation is progressive, characterized by continuous rather than discrete changes. Cells at different developmental stages express distinct gene sets. With scRNA-seq, we can capture cells at various stages, enabling exploration of continuous differentiation trajectories. The UMAP dimensionality reduction algorithm outperforms other algorithms in capturing the global and topological structures of datasets, including the spatial positioning of individual cells. We employed the slingshot R package to assess cellular differentiation processes. The trajectory analysis identified CD4_Naïve as the starting point, culminating in CD8_CM, suggesting CD8_CM as terminally differentiated CD8_T cells (Fig. 6a). To discern the role of CD8_CM in both COVID-19 and LUAD and its intercellular relationships, we conducted cellular communication analysis. By analyzing samples from COVID-19 and LUAD, we constructed a communication network of CD8_CM with other cells (Fig. 6b, d). Comparative analysis of pathways enriched in CD8_CM and other cells revealed frequent communication through MIF -(CD74 + CD44) and MIF -(CD74 + CXCR4) signals with B cells, NK cells, and CD8_EM cells in both diseases (Fig. 6c, e). Thus, CD8_CM is posited as a pivotal player in both COVID-19 and LUAD.

### MR analysis using key marker genes identifies three novel causal genes for LUAD

**Fig. 7:**
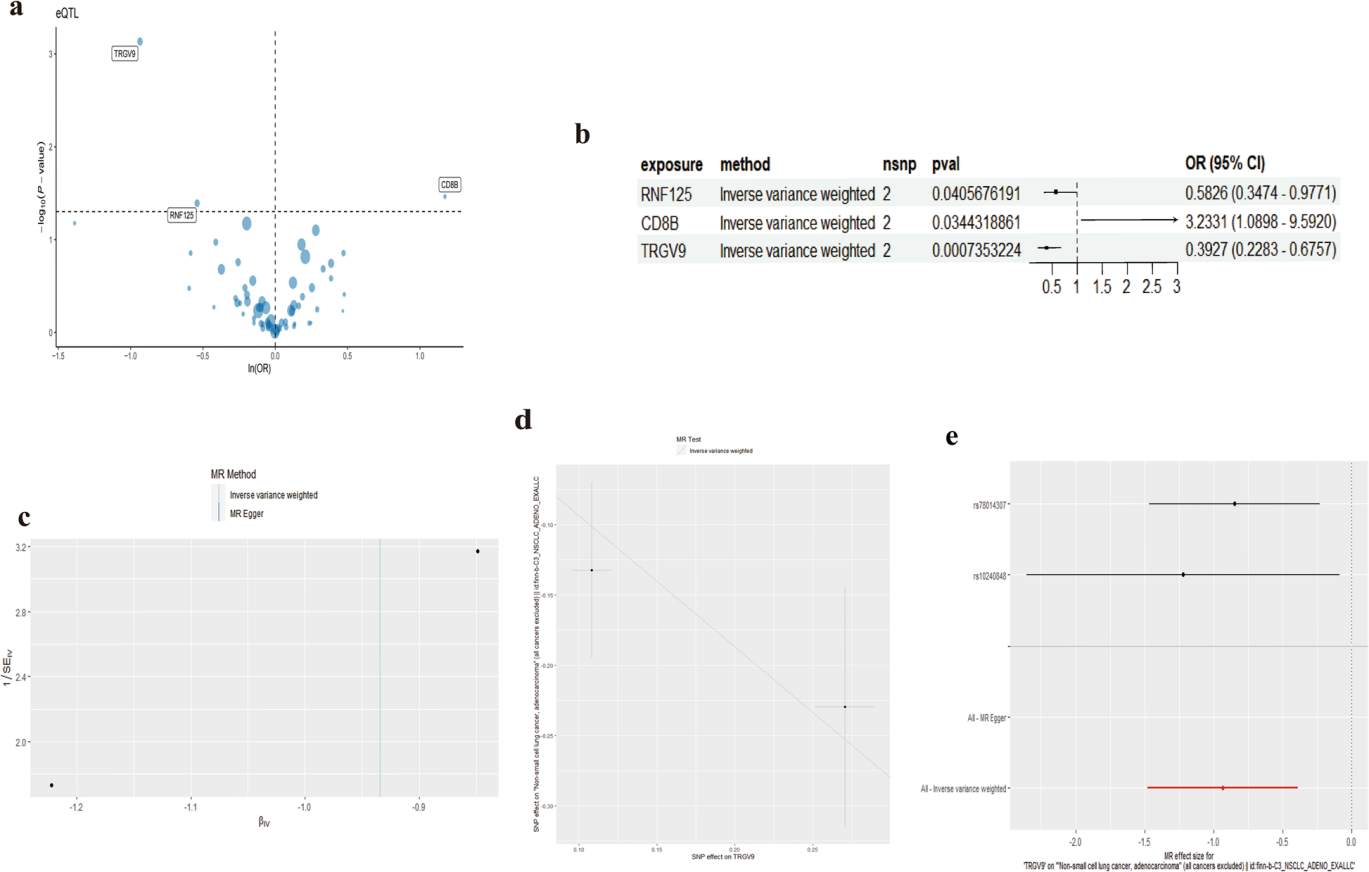
**a** Volcano plot illustrating the association between key genes and the risk of lung adenocarcinoma. **b** Forest plot depicting the association of key genes with the risk of lung adenocarcinoma. **c** Mendelian Randomization Heterogeneity Test This assesses the consistency of genetic associations across different SNPs. **d** Pleiotropy Test Evaluates the horizontal pleiotropy across various key genes. **e** Validity and Robustness of Mendelian Randomization. **f** Results Further validates the identified associations, indicating minimal bias and confounding

We conducted Mendelian randomization (MR) analysis on single-cell RNA sequencing data for lung cancer to uncover key genes potentially influencing lung adenocarcinoma risk. Initially, using the Seurat tool, we identified 70 key genes (Supplementary file 1) distinguishing CD8_CM T cells from other cell types and subtypes and determined significant markers for subsequent analysis. For deeper insight, we successfully converted gene symbols to corresponding ENSEMBL IDs using org.Hs.eg.db. This was followed by a two-sample MR analysis, involving extraction of SNP data related to our genes of interest as exposure data, retrieving lung adenocarcinoma outcome data from the EBI database, and harmonizing between exposure and outcome datasets. The MR analysis highlighted several genes significantly associated with lung cancer risk. Specifically, the RNF125 gene exhibited an odds ratio (OR) of 0.5826 (95% CI: 0.3474—0.9771, *p* = 0.0406). For the CD8B gene, the OR was 3.2331 (95% CI: 1.0898—9.5920, *p* = 0.0344). The TRGV9 gene displayed an OR of 0.3927 (95% CI: 0.2283—0.6757, *p* = 0.0007). These associations hint at potential genetic markers influencing the aforementioned cancer risk. To visually represent the -log10-transformed *p*-values against ln (OR) for each gene, we generated a volcano plot, with genes showing significant *p*-values highlighted (Fig. 7a). The plot distinctly reveals genes with significant positive and negative associations. Subsequently, we developed a forest plot, visualizing the OR and 95% CI for each significant gene, emphasizing the robustness and direction of each gene's association (Fig. 7b, c, d and e). In the two-sample Mendelian randomization (MR) analysis, it was observed that RNF125, CD8B, and TRGV9 exhibit no heterogeneity concerning lung adenocarcinoma (LUAD). The results are detailed in (Supplement Table 2). Employing the Inverse Variance Weighted method, the analysis revealed that RNF125, CD8B, and TRGV9 do not exhibit horizontal pleiotropy in the MR analysis of LUAD (RNF125: *P* = 0.755703216; CD8B: *P* = 0.711380024; TRGV9: *P* = 0.571487335) (Supplement Table 3, Supplement Table 1). These tests evaluated the consistency of genetic associations across different SNPs and identified potential horizontal pleiotropy. The analysis validated our identified associations, indicating minimal biases and confounding. The results of this study offer fresh perspectives and directions for subsequent biological validation and mechanistic research.

### Mendelian randomization and colocalization analysis of genes RNF125, CD8B and TRGV9 with lung cancer risk

**Fig. 8:**
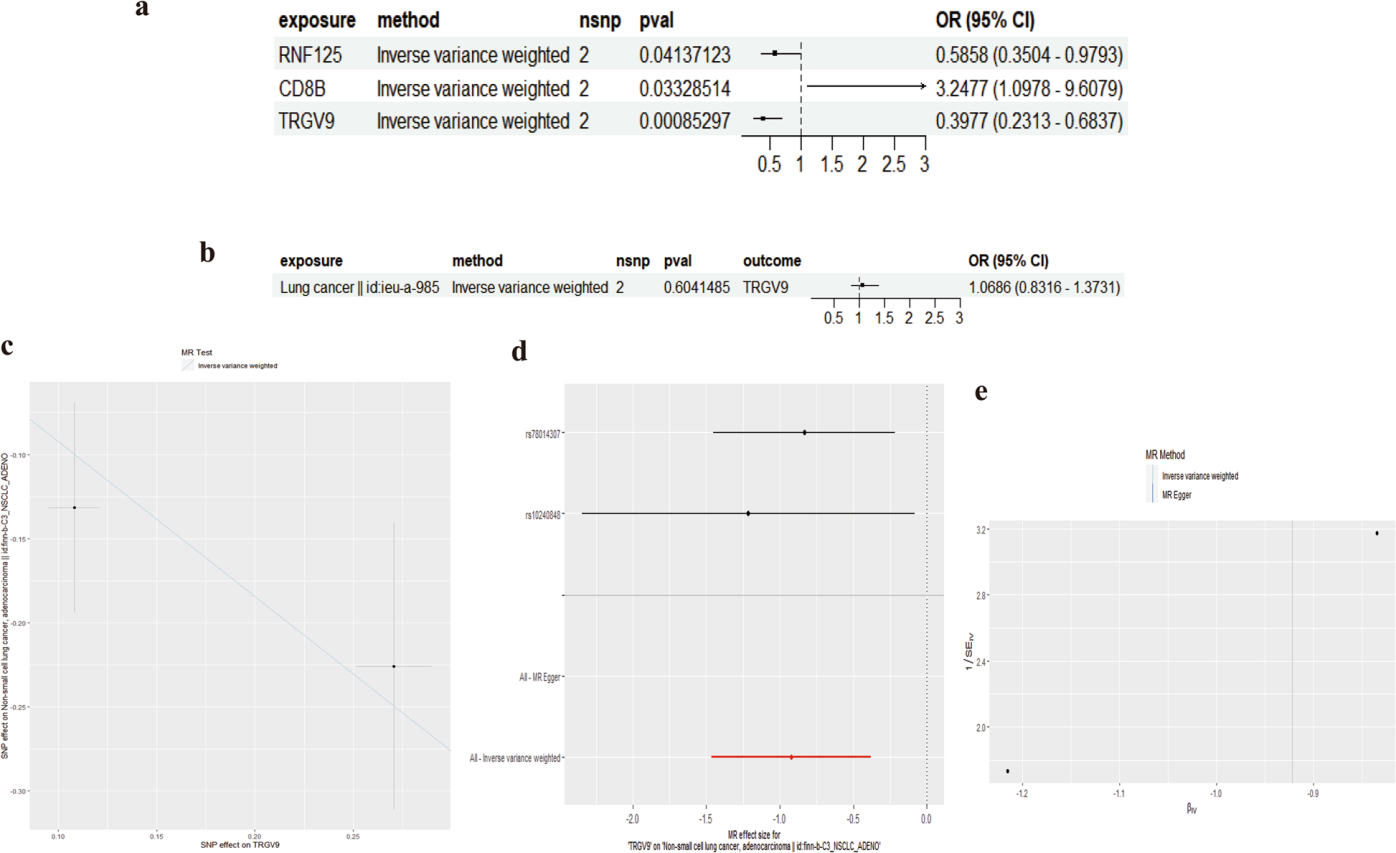
Validation Queue, Bidirectional Mendel. **a** Mendelian randomization analysis showing associations of RNF125, CD8B, and TRGV9 gene variants with lung cancer risk. **b** Reverse Mendelian randomization depicting the relationship between lung cancer and the TRGV9 gene. **c** Scatter plot examining heterogeneity in the Mendelian randomization analysis. **d** Forest plot illustrating the effect sizes for the associations. **e** Funnel plot assessing potential pleiotropy in the Mendelian randomization results

To verify the reliability of our findings, we conducted a validation set analysis using Mendelian randomization. We explored the association between three genes, namely RNF125, CD8B, and TRGV9, and lung cancer risk. The results showed a significant association of RNF125 mutations with lung cancer risk (OR = 0.5858, 95% CI: 0.3504–0.9793). Mutations in CD8B were associated with an increased lung cancer risk (OR = 3.2477, 95% CI: 1.0978–9.6079), while mutations in TRGV9 were linked to a reduced risk (OR = 0.3977, 95% CI: 0.2313–0.6837) (Fig. 8a). Additionally, the MR analysis for lung cancer and the TRGV9 gene showed a non-significant association (OR = 1.0686, 95% CI: 0.8316–1.3731). We also conducted a reverse MR analysis, which, when considering lung cancer as the exposure and the TRGV9 gene as the outcome, did not show a significant association (Fig. 8b, c, d and e).

### Investigating the association between the TRGV9 gene and lung adenocarcinoma using Mendelian Randomization

**Fig. 9:**
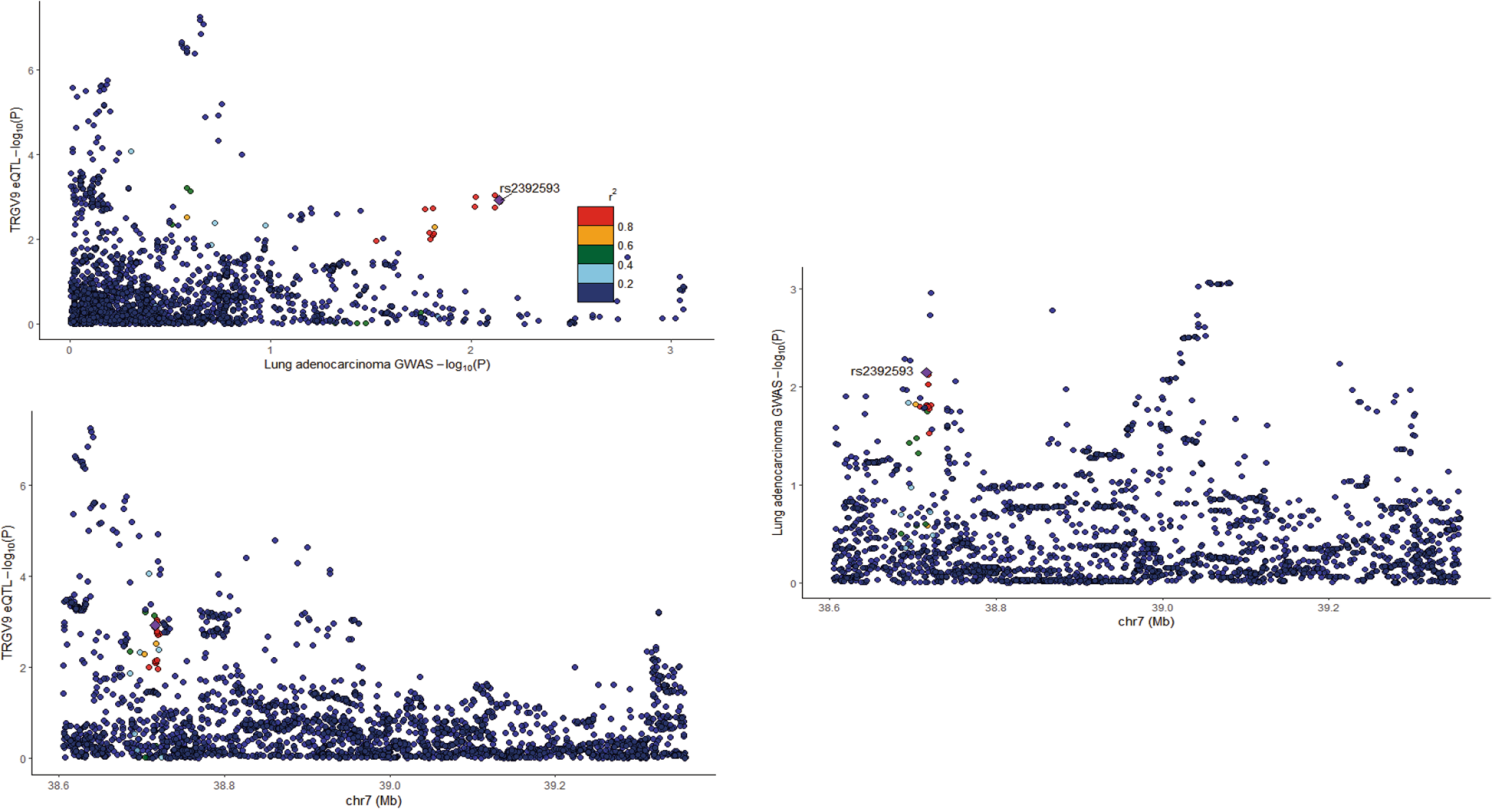
Regional association map

In this study, we employed the Mendelian randomization approach to probe the association between the TRGV9 gene and lung adenocarcinoma. Initially, we displayed the regional association plots for the TRGV9 gene eQTL in tandem with the lung adenocarcinoma GWAS results. By contrasting their association strengths, we identified several single nucleotide polymorphisms (SNPs) demonstrating pronounced associations across both phenotypes. Notably, specific SNPs, such as rs23923593, associated with the TRGV9 gene eQTL, also exhibited significant correlation in the lung adenocarcinoma GWAS. This provided preliminary evidence hinting at a potential link between this gene and lung adenocarcinoma development (Fig. 9). We employed the Phenoscanner tool to further delve into these SNPs' associations with other traits. The results revealed associations of some SNPs with diverse traits, suggesting their involvement in various biological processes. To ascertain the causative direction in our MR analysis, we performed the Steiger test, which discerns which trait lies closer to the gene and hence is more likely the true exposure. Our findings predominantly aligned with our preliminary hypothesis, suggesting that alterations in TRGV9 expression could modulate lung adenocarcinoma risk. Collectively, our analyses offer preliminary evidence supporting a potential causal relationship between the TRGV9 gene and lung adenocarcinoma.

### Single-cell transcriptomic analysis reveals TRGV9's crucial role in T-cell metabolism and function in LUAD

**Fig. 10:**
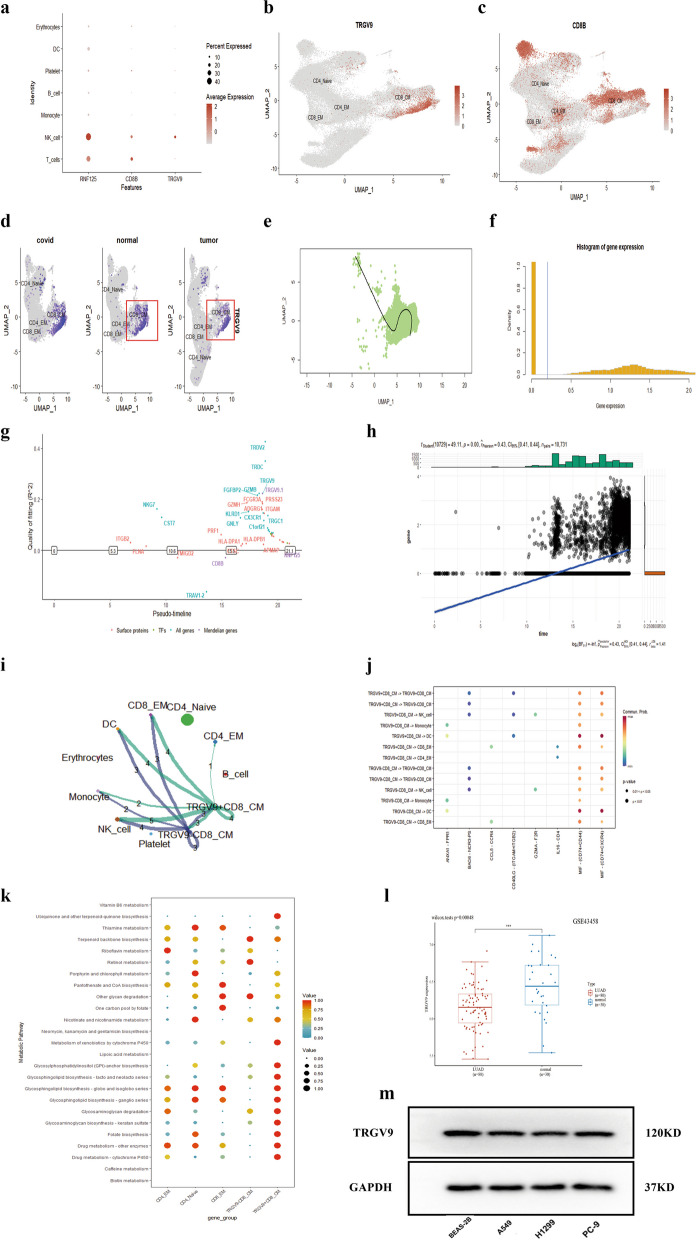
Single-cell transcriptomic analysis reveals the pivotal role of TRGV9 in T-cell metabolism and function in LUAD: **a** Expression pattern of key genes across different cell subpopulations. **b** UMAP dimensionality reduction visualizing cell subgroups with differential expressions of specific key genes. **c** Expression distribution of the CD8B gene across cells plotted using UMAP. **d** UMAP plots showing the distribution of cells in conditions of COVID, normal, and tumor, highlighting the expression of TRGV9 and CD8B. **e** Cell trajectory analysis from primary to mature T-cell states using Slingshot. **f** Histogram representing the distribution of gene expression across cells. **g** Gene on/off status in pseudo-time, reflecting gene expression dynamics during cellular developmental or transcriptional processes. **h** Scatter plot depicting the relationship between the expression of the 'TRGV9' gene and pseudo-time. **i** Intercellular communication analysis between TRGV9 + CD8_CM and TRGV9-CD8_CM cell subgroups using the CellChat tool. **j** Differential signaling pathways enriched in communications involving TRGV9 + CD8_CM cells compared to others. **k** Metabolic pathway analysis showing the activation states of T cells, revealing differences in metabolic activities between TRGV9 + CD8_CM and TRGV9-CD8_CM cell subgroups. **l** Differential gene expression analysis between TRGV9 + CD8_CM and TRGV9-CD8_CM cell subpopulations, with a focus on the significant difference in TRGV9 expression between LUAD and healthy groups. **m** Expression of TRGV9 in normal cells and three adenocarcinoma cell types

Utilizing the UMAP algorithm, we performed dimensionality reduction and visualization on single-cell transcriptomic data to identify distinct cellular subpopulations. We first visualized the expression patterns of three key marker genes across different cell clusters (Fig. 10a). Subsequently, we employed the feature plot function to specifically highlight the expression of TRGV9 and CD8B within individual cells (Fig. 10b and c). Moreover, we segmented the data based on tissue type and conducted a focused analysis of TRGV9 expression, revealing a lower level of expression in tumor tissues compared to normal tissues (Fig. 10d), thus providing clues for further functional studies. Employing the Slingshot tool, we performed cell trajectory analysis, observing the transition of T cells from a naive to a mature state along the trajectory (Fig. 10e), which suggests potential dynamic changes of T cells during disease progression. The distribution of gene expression is shown in Fig. 10f, where most genes displayed low activity levels, while a minority exhibited relatively high expression. To explore changes in gene expression during cell development or transcriptional dynamics, we depicted the on/off states of various genes over pseudotime (Fig. 10g). Scatter plot analysis was used to depict the relationship between the expression of the 'TRGV9' gene and pseudotime. Statistical analysis indicated a moderate positive correlation (Pearson *r* = 0.43, *p* < 0.001), suggesting that 'TRGV9' expression might increase over pseudotime (Fig. 10h). Intercellular communication analysis, conducted with the CellChat tool, showed differences between the TRGV9 + CD8_CM and TRGV9-CD8_CM cell subgroups (Fig. 10i), with the TRGV9 + CD8_CM subgroup demonstrating more ligand-receptor interactions and stronger cell signaling activities. Furthermore, by comparing enriched signaling pathways between TRGV9 + CD8_CM cells and other cells, we noted that the MIF—(CD74 + CD44) and MIF—(CD74 + CXCR4) signaling pathways were involved in cell communication in LUAD, paralleling our previous findings (Fig. 10j). Metabolic pathway analysis revealed distinct states of T cell activation; using the scMetabolism tool, we assessed metabolic activities within the cells. Notably, key metabolic pathways differed significantly between the TRGV9 + CD8_CM and TRGV9-CD8_CM subgroups. Specifically, the TRGV9 + CD8_CM subgroup exhibited enhanced uric acid synthesis capabilities, potentially linked to rapid T cell division and energy requirements. In the tricarboxylic acid (TCA) cycle, TRGV9 + CD8_CM cells showed increased activity, suggesting higher oxidative phosphorylation and ATP production. Compared to the TRGV9-CD8_CM subgroup, TRGV9 + CD8_CM cells displayed reduced fatty acid oxidation activity, likely reflecting a preference for glucose over fatty acids as an energy source. Additionally, the TRGV9 + CD8_CM subgroup showed increased nucleotide synthesis activity, consistent with rapid proliferation and DNA synthesis demands. These differences could be related to T cell activation, proliferation, and immune functions (Fig. 10k), providing important insights for further research. Differential gene expression analysis between TRGV9 + CD8_CM and TRGV9-CD8_CM cell subgroups revealed several genes differentially expressed in association with LUAD. Notably, the TRGV9 gene exhibited significant differences between LUAD and healthy groups. Similar gene expression patterns were observed in the overall transcriptomic data (Fig. 10l), further validating the accuracy and reliability of our single-cell analysis. Additionally, partial biological validation was performed, with Western blotting analysis indicating higher expression of TRGV9 in a normal cell line compared to three other lung adenocarcinoma cell lines (Fig. 10m), corroborating the results from our dataset analysis.

### Trajectory inference of T cell subsets

We conducted cell trajectory analysis to infer the differentiation status of T-cell subtypes, understanding the evolutionary process of cells in LUAD, while simultaneously demonstrating the trajectories of different differential genes. (Fig. S4a) illustrates pseudo-temporal trajectories of four T-cell subtypes. The specific trajectory directions are depicted in (Fig. S4b), where the analysis reveals that CD4_Naïve serves as the pseudo-temporal starting point, while CD8_CM represents the endpoint of cell pseudo-temporality. Additionally, we present the expression status of the top 10 differential genes in different cell subtypes (Fig. S4c), as well as the pseudo-temporal expression changes of the top 10 genes across different cells (Fig. S4d). Finally, we identified 240 co-expression gene modules (Fig. S4e).

### Correlation analysis of TRGV9 expression differences with clinical features

Initially, utilizing the GeneCards website [27] (https://www.genecards.org/), we identified TRGV9, also known as TRGC1. Through an analysis of TCGA-LUAD and its associated clinical information, we observed higher expression of TRGV9 in normal tissues compared to tumor tissues (Fig. S5a). Additionally, TRGV9 exhibited correlation with prognosis (*p* = 0.001) (Fig. S5b). Subsequently, we investigated the correlation of TRGV9 with clinical features.We assessed the correlation between TRGV9 expression and clinical features using a heatmap. The results revealed significant differences in the distribution of N staging, tumor grading, and T staging between high and low expression groups (Fig. S5c). Specifically, significant differences were observed between N0 and N1 (*p* = 0.024) and N0 and N1 (0.00069) (Fig. S5d). Furthermore, significant differences existed between stage I and stage III (*p* = 0.001) (Fig. S5e). Lastly, we identified significant differences between T1 and T2 (*p* = 0.0021) as well as between T1 and T3 (*p* = 0.038) within the T staging (Fig. S5f). In summary, the expression of TRGV9 appears to be closely associated with the clinical prognosis of patients with lung adenocarcinoma.

## Discussion

The advent of single-cell RNA sequencing (scRNA-seq) technology has furnished a unique opportunity to elucidate transcriptional heterogeneity among diverse cell types under various disease conditions, which is instrumental for a deeper understanding of cellular functions and interactions. In this study, scRNA-seq analysis was employed to explore the disparities among cell sub-populations between COVID-19, lung adenocarcinoma (LUAD), and healthy controls, with a particular focus on T cells.

CD8_CM cells, a sub-group within the CD8 + T cell lineage, are memory T cells formed post-antigen activation [28]. Upon re-encounter with the corresponding antigen, CD8_CM cells proliferate rapidly and differentiate into effector T cells, playing a vital role in secondary immune responses. They are predominantly found in peripheral blood and lymphatic tissues [29], exhibiting both memory and effector functionalities, thereby constituting a crucial component of cellular immune memory. They play a pivotal role in antiviral and antitumor immunity [30, 31]. CD8 + T cells, especially the CD8_CM subset, are primary tumor-infiltrating immune cells responsible for delivering antitumor responses. Immunotherapeutic strategies aiming to restore the effector function of CD8 + T cells have provided support for the current successful cancer immunotherapy [32, 33]. Long-lived memory CD8 + T cells play an important role in tumor immunity, inclusive of central, effector, stem-like, and tissue-resident memory CD8 + T cell subsets [34]. Lung cancer induces functional defects in CD8 + T cells, which correlates with the clinical response to immunotherapy. A deeper understanding of the impact of lung cancer on CD8 + T cells might aid in the development of new therapeutic approaches [35]. Research indicates that SARS-CoV-2 mRNA vaccines can offer protection against severe disease as early as ten days post-vaccination, a time when neutralizing antibodies are barely detectable, suggesting that vaccine-induced CD8 + T cells might be the main mediators of protection during this early stage [36]. CD8 + T cells are essential for protective immune responses, directly participating in viral clearance, thereby playing a significant role in viral infections, including SARS-CoV-2 [30]. It was discovered that CD8 + T cell responses are robustly activated one week post bnt162b2 mRNA vaccination, at a time when circulating CD4 + T cells and neutralizing antibodies are only weakly detectable [36]. CD8 + T cells might be the principal protective mediators induced by mRNA vaccines, expanding in the early protection window post prime vaccination, preceding the maturation of other effector arms of vaccine-induced immunity, and are stably maintained post boost vaccination [36]. Particularly, CD8_CM cells play a significant role in immune surveillance and antitumor immune responses in lung adenocarcinoma [37]. In patients with lung adenocarcinoma, defective CD8_CM cells are present; although these cells exhibit a memory T cell phenotype, their functionality resembles effector T cells, manifesting weakened effector function and reduced proliferative capacity [38]. Additionally, the number of CD8_CM cells in lung adenocarcinoma tissues is lower compared to peripheral blood, and their cytokine secretion ability is diminished. This can be partly attributed to the tumor microenvironment inhibiting CD8_CM cell activity through mechanisms like the PD-1/PD-L1 pathway [39]. Enhancing the infiltration and activity of CD8_CM cells in lung adenocarcinoma tissues through immunotherapeutic means is a crucial approach to augment antitumor immune responses in patients with lung adenocarcinoma [40]. Some studies have shown that PD-1/PD-L1 inhibitors can partially restore the functionality of CD8_CM cells in tumors [41–44]. The subset and functional state of CD8_CM cells can serve as significant biomarkers for predicting and monitoring the therapeutic efficacy of immunotherapy for lung adenocarcinoma [45]. Optimizing the functionality of CD8_CM cells will contribute to further advancements in immunotherapy for lung adenocarcinoma. Moreover, CD8_CM cells are key effector cells against SARS-CoV-2 infection, rapidly proliferating and differentiating into cytotoxic T lymphocytes in COVID-19 patients, directly recognizing and killing virus-infected cells, thus inhibiting viral replication [46, 47]. The increase in CD8_CM cell numbers in COVID-19 patients correlates with the severity of the disease, indicating their involvement in the body's antiviral immune responses. Monitoring the dynamic changes of CD8_CM cells can assess the immune status of the body, providing a basis for evaluating the effects of subsequent immunotherapy and vaccination. In summary, the subset and functional state of CD8_CM cells are crucial for immune responses, whether in tumor immunity or in combating SARS-CoV-2 and other viral infections. In-depth exploration of the biological characteristics and functions of CD8_CM cells, along with optimizing their functionality through immunotherapeutic strategies, will offer significant insights for advancing both antitumor and antiviral immunotherapy.

This study systematically investigated the composition and functionality of immune cells in the peripheral blood of COVID-19 and LUAD patients, as well as healthy individuals, through scRNA-seq analysis. Our data initially validated the presence of T cells, B cells, NK cells, etc., in peripheral blood, consistent with existing literature [48]. Notably, compared to healthy controls, both COVID-19 and LUAD patient groups exhibited a varying degree of decrease in the proportion of mature effector CD8 + T cells (CD8_CM). This might be associated with weakened immune function under disease conditions. For instance, multiple research groups have validated the reduction in CD8 + T cell numbers and cytotoxicity in the peripheral blood of COVID-19 patients [49, 50]. Moreover, in mouse tumor models, the tumor microenvironment has been shown to inhibit the proliferation of CD8 + T cell [51]. Therefore, the variation in CD8_CM cell proportions may reflect their significant role in disease immune responses. Through functional enrichment analysis, we discovered characteristic pathways of different cell sub-groups correlating with known cellular functions, providing clues to understand the physiological functions of each cell type. Additionally, through single-cell trajectory analysis, we unveiled the dynamic differentiation process of T cells from early to mature stages, offering a new perspective for studying T cell development and activation. Furthermore, cell–cell communication network analysis revealed enhanced connections between CD8_CM T cells and B cells, NK cells, etc., suggesting their potential involvement in regulating other immune cells during disease progression. This study not only identified disease-related immune cell alterations but also proposed potential molecular mechanisms through multi-omics analysis. For example, we found that the reduction in TRGV9 expression might be one of the causes leading to CD8 + T cell functional decline. TRGV9 is a T cell receptor variable region gene involved in T cell antigen recognition [52]. In the tumor microenvironment, suppression of TRGV9 expression results in T cells failing to recognize tumor antigens, thereby reducing their effector functionality. In fact, in mouse tumor models, overexpression of TRGV9 enhances T cell cytotoxicity against tumor [53]. Hence, modulating TRGV9 expression levels might emerge as a new strategy to boost T cell antitumor effects. Additionally, RNF125, a ubiquitin ligase, also participates in various signaling pathway processes, and the specific mechanisms related to diseases require further investigation [54]. The expression level of TRGV9 in lung adenocarcinoma patients is closely associated with clinical features, where higher expression correlates with a better prognosis. The clinical relevance suggests significant differences in the pathological characteristics of lung adenocarcinoma, particularly in tumor staging and grading. These findings imply that TRGV9 could serve as a potential prognostic marker, offering valuable leads for further in-depth research and clinical applications in the future.

It's imperative to acknowledge certain limitations in this study. Firstly, due to the limited sample size, the results necessitate validation through an expanded sample scale. Moreover, subsequent studies are required to experimentally validate the functional roles of the predicted key genes in disease onset. Animal model studies should also be conducted to explore the molecular mechanisms and their regulatory modes. Besides, scRNA-seq technology has its inherent batch effects, which require caution in data interpretation. Technical noise during sample processing and library construction in scRNA-seq could lead to batch effects, hence careful interpretation of results is essential. Future research needs to increase the sample size and adopt more standardized procedures to validate the findings. Secondly, Mendelian randomization analysis relies on the correlation between genetic markers and phenotypes to infer causal relationships. However, potential genetic pleiotropy might lead to false-positive results. Thus, the newly discovered candidate genes still require functional validation.

## Conclusion

This study preliminarily identified the association of immune cell composition and key gene expression with COVID-19 and LUAD, proposing some verifiable hypotheses. This lays the groundwork for exploring the molecular mechanisms of diseases, and also provides potential targets for the development of new preventive and therapeutic strategies. Subsequent research should employ a variety of experimental methods for validation and exercise caution in interpreting the biological significance of various omics data. This study offers valuable experience in bridging the gap between single-cell omics and genetics, and serves as a paradigm for precision medicine research in complex diseases.

### Supplementary Information

**Additional file 1: Fig. s1.** Analysis ofRNA-sequencing ata: a. Distribution of RNA features across different samples: nFeature RN: Number of unique RNA features in the samples. nCount RNA: Total count of RNA molecules in the samples, percent.mt: Percentage of mitochondral genes in the samples, percent-HB: Percentage of HB genes in the samples. b. Cell distribution visualized through tsne and UMAP algorithms within 9 samples before harmony. c. Cell distribution visualized through tsne and UMAP algorithms within 9 samples after harmony D. The relationship between "harmony" and "Standard Deviation".

**Additional file 2: Fig. s2.** For validation and to ensure the reliability of the annotations, manual annotations were also performed. The expression patterns of specific marker genes for each cell type are represented through violin plots and feature plots.

**Additional file 3: Fig. s3.** a. T_cell distribution visualized through tsne and UMAP algorithms within 9 samples before harmony. b. The relationship between "harmony" and "Standard Deviation"before harmony c. T_cell distribution visualized through tsne and UMAP algorithms within 9 samples after harmony d. The relationship between "harmony" and "Standard Deviation" after harmony.

**Additional file 4.**

**Additional file 5.**

**Additional file 6.**

**Additional file 7: Fig. s4.** Simulated time-series analysis a. Time trajectories of CD4_Naïve, CD4_EM, CD8_CM, and CD8_EM b. Developmental direction of cells over time c. Feature plot of 10 genes across 4 T cell subgroups d. Simulated time-series trajectories of differential genes.** Fig. s5.** Correlation analysis between differential TRGV9 expression and clinical features a. Analysis of TRGV9 expression differences between normal tissues and LUAD b. Survival analysis of TRGV9 in LUAD c. Heatmap illustrating the association of TRGV9 expression with age, gender, stage, T, and N of LUAD patients d. Correlation of TRGV9 expression with N staging e. Correlation of TRGV9 expression with stage f. Correlation of TRGV9 expression with T staging.** Supplement Table 1.** Impact of RNF125, CD8B, and TRGV9 on lung adenocarcinoma.

## Data Availability

This study analyzed publicly available data sets. All original analytical datasets used or analyzed during the current study are available from the corresponding author on reasonable request.
