# Supplementary material for: Integrative single-cell analysis: dissecting CD8 + memory cell roles in LUAD and COVID-19 via eQTLs and Mendelian Randomization
Source: Hereditas. 2024 Jan 31;161:7. doi: 10.1186/s41065-023-00307-7 (PMC10829297; doi:10.1186/s41065-023-00307-7)
Supplement: Supplementary file 1 — Additional file 1: Fig. s1. Analysis ofRNA-sequencing ata: a. Distribution of RNA features across different samples: nFeature RN: Number of unique RNA features in the samples. nCount RNA: Total count of RNA molecules in the samples, percent.mt: Percentage of mitochondral genes in the samples, percent-HB: Percentage of HB genes in the samples. b. Cell distribution visualized through tsne and UMAP algorithms within 9 samples before harmony. c. Cell distribution visualized through tsne and UMAP algorithms within 9 samples after harmony D. The relationship between "harmony" and "Standard Deviation". [file 41065_2023_307_MOESM1_ESM.pdf]

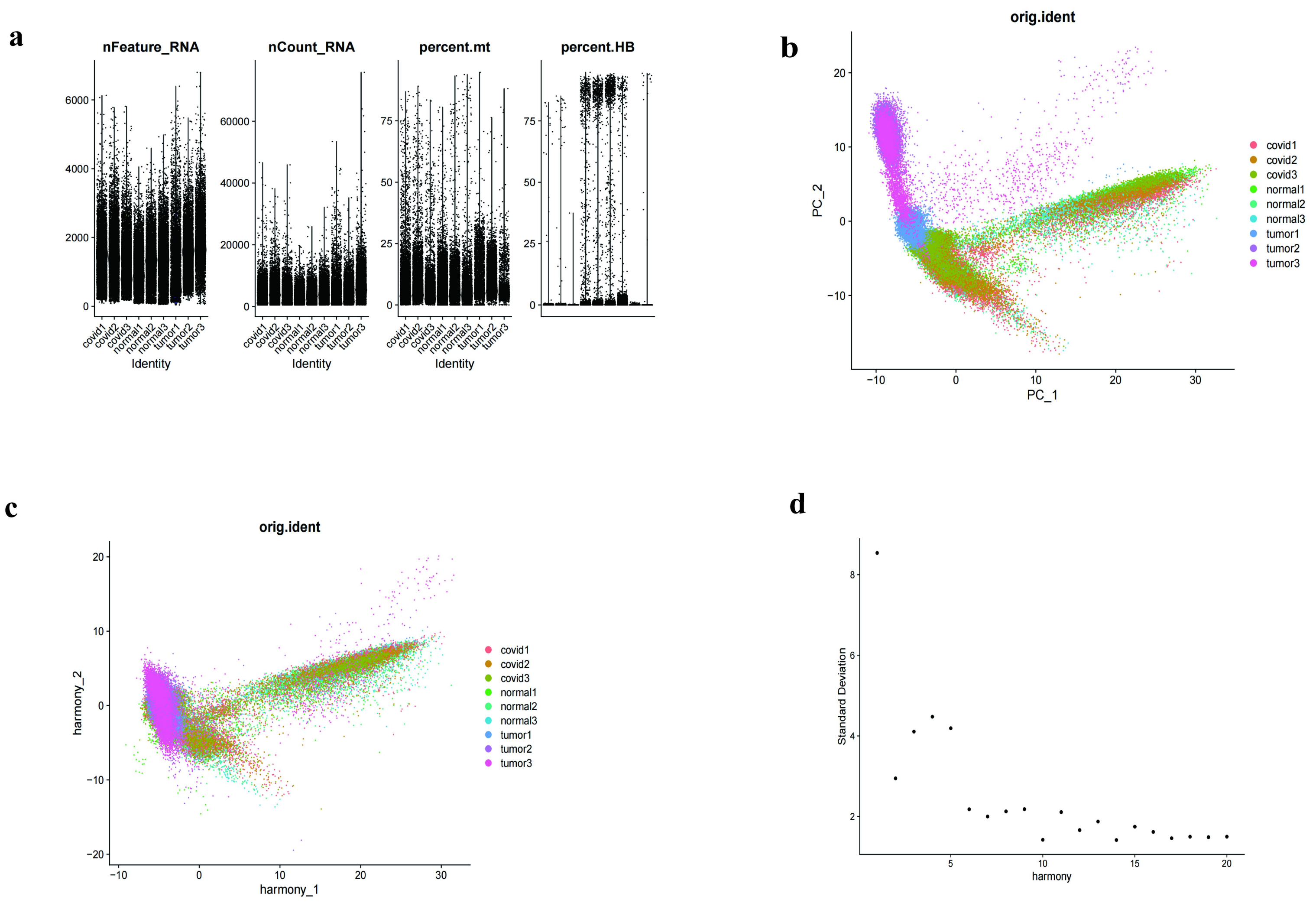

Fig s1 Analysis ofRNA-sequencing ata: **a**. Distribution ofRNA features across diferent samples:nFeature RN: Number of unique RNA features in thesamples. nCount RNA: Total count ofRNA molecules in the samples,percent.mt: Percentage of mitochondral genes in the samples,percent-HB: Percentage ofHBgenes in the samples. **b**. Cell distribution visualized through tsne and UMAP algorithms within 9 samples before harmony. **c**. Cell distribution visualized through tsne and UMAP algorithms within 9 samples after harmony D.The relationship between "harmony" and "Standard Deviation"
