# Supplementary material for: Integrative single-cell analysis: dissecting CD8 + memory cell roles in LUAD and COVID-19 via eQTLs and Mendelian Randomization
Source: Hereditas. 2024 Jan 31;161:7. doi: 10.1186/s41065-023-00307-7 (PMC10829297; doi:10.1186/s41065-023-00307-7)
Supplement: Supplementary file 2 — Additional file 2: Fig. s2. For validation and to ensure the reliability of the annotations, manual annotations were also performed. The expression patterns of specific marker genes for each cell type are represented through violin plots and feature plots. [file 41065_2023_307_MOESM2_ESM.pdf]

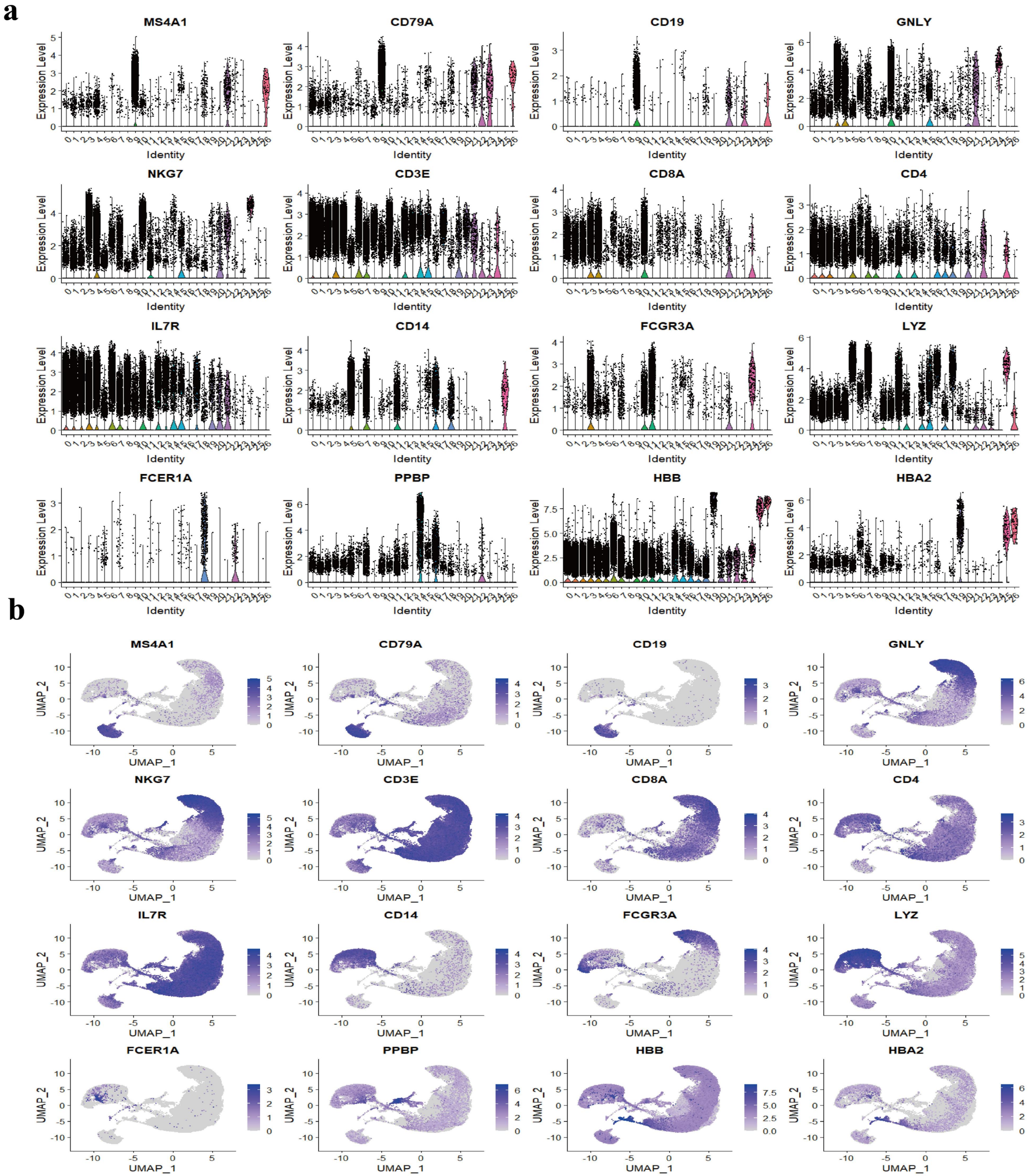

Fig s2 For validation and to ensure the reliability of the annotations, manual annotations were also performed. The expression patterns of specific marker genes for each cell type are represented through violin plots and feature plots.
