# Supplementary material for: Integrative single-cell analysis: dissecting CD8 + memory cell roles in LUAD and COVID-19 via eQTLs and Mendelian Randomization
Source: Hereditas. 2024 Jan 31;161:7. doi: 10.1186/s41065-023-00307-7 (PMC10829297; doi:10.1186/s41065-023-00307-7)
Supplement: Supplementary file 3 — Additional file 3: Fig. s3. a. T_cell distribution visualized through tsne and UMAP algorithms within 9 samples before harmony. b. The relationship between "harmony" and "Standard Deviation"before harmony c. T_cell distribution visualized through tsne and UMAP algorithms within 9 samples after harmony d. The relationship between "harmony" and "Standard Deviation" after harmony. [file 41065_2023_307_MOESM3_ESM.pdf]

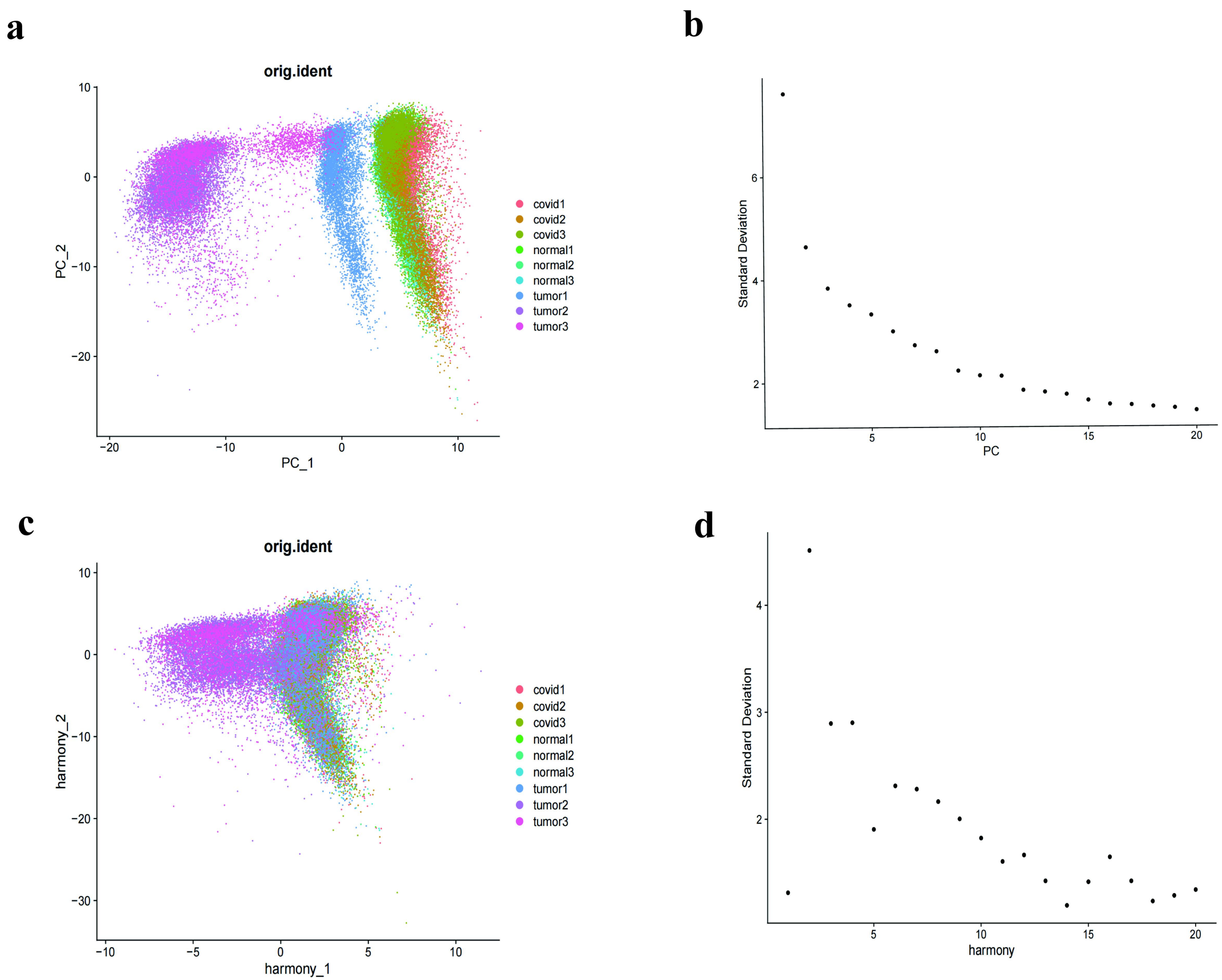

Fig s3 **a**. T_cell distribution visualized through tsne and UMAP algorithms within 9 samples before harmony. **b.**The relationship between "harmony" and "Standard Deviation"before harmony **c.** T_cell distribution visualized through tsne and UMAP algorithms within 9 samples after harmony **d.**The relationship between "harmony" and "Standard Deviation" after harmony.
