# Supplementary material for: Integrative single-cell analysis: dissecting CD8 + memory cell roles in LUAD and COVID-19 via eQTLs and Mendelian Randomization
Source: Hereditas. 2024 Jan 31;161:7. doi: 10.1186/s41065-023-00307-7 (PMC10829297; doi:10.1186/s41065-023-00307-7)
Supplement: Supplementary file 7 — Additional file 7: Fig. s4. Simulated time-series analysis a. Time trajectories of CD4_Naïve, CD4_EM, CD8_CM, and CD8_EM b. Developmental direction of cells over time c. Feature plot of 10 genes across 4 T cell subgroups d. Simulated time-series trajectories of differential genes. Fig. s5. Correlation analysis between differential TRGV9 expression and clinical features a. Analysis of TRGV9 expression differences between normal tissues and LUAD b. Survival analysis of TRGV9 in LUAD c. Heatmap illustrating the association of TRGV9 expression with age, gender, stage, T, and N of LUAD patients d. Correlation of TRGV9 expression with N staging e. Correlation of TRGV9 expression with stage f. Correlation of TRGV9 expression with T staging. Supplement Table 1. Impact of RNF125, CD8B, and TRGV9 on lung adenocarcinoma. [file 41065_2023_307_MOESM7_ESM.pdf]

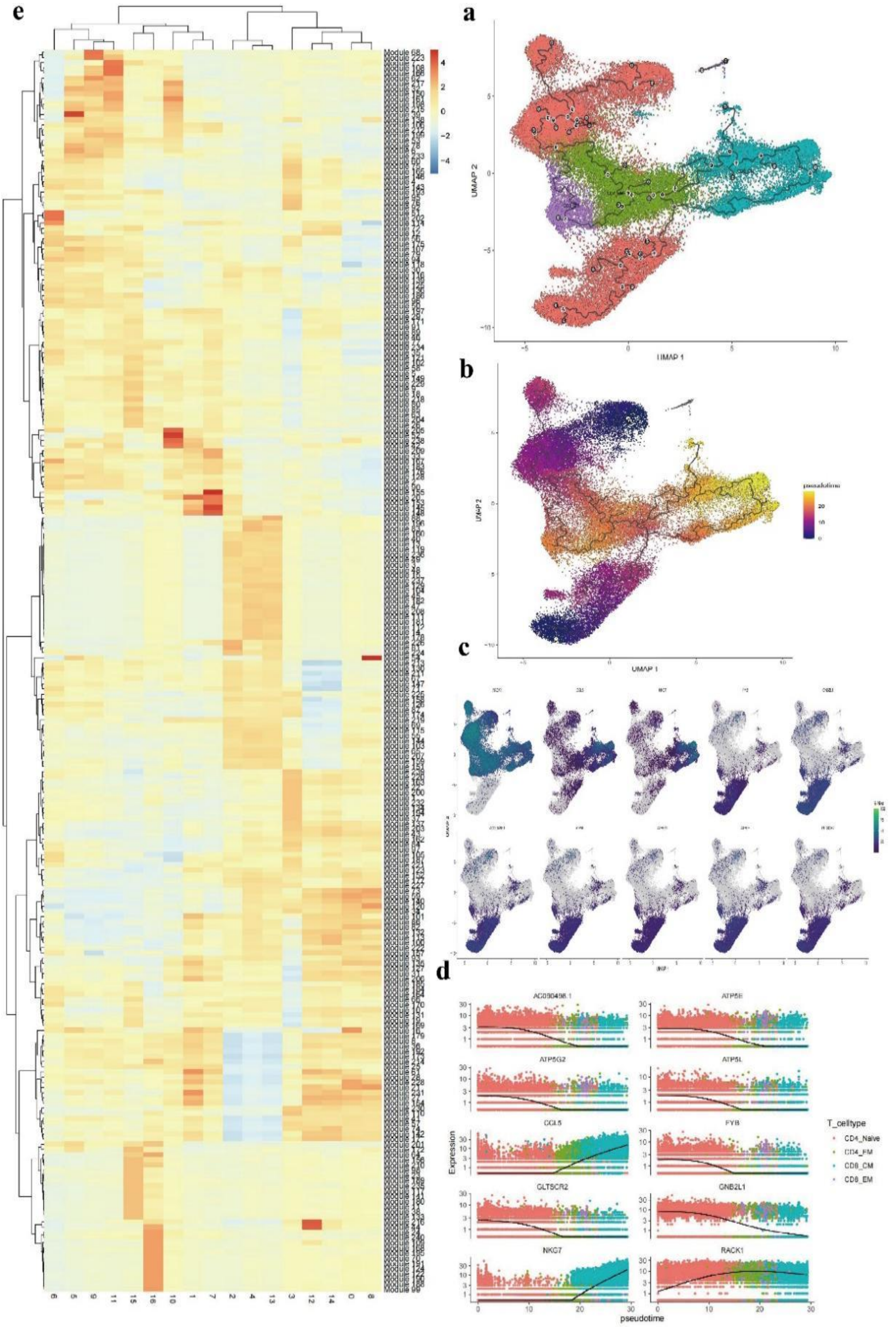

Fig.s4 Simulated time-series analysis a. Time trajectories of CD4_Naïve, CD4_EM, CD8_CM, and CD8_EM **b.** Developmental direction of cells over time **c.** Feature plot of 10 genes across 4 T cell subgroups **d.** Simulated time-series trajectories of differential genes

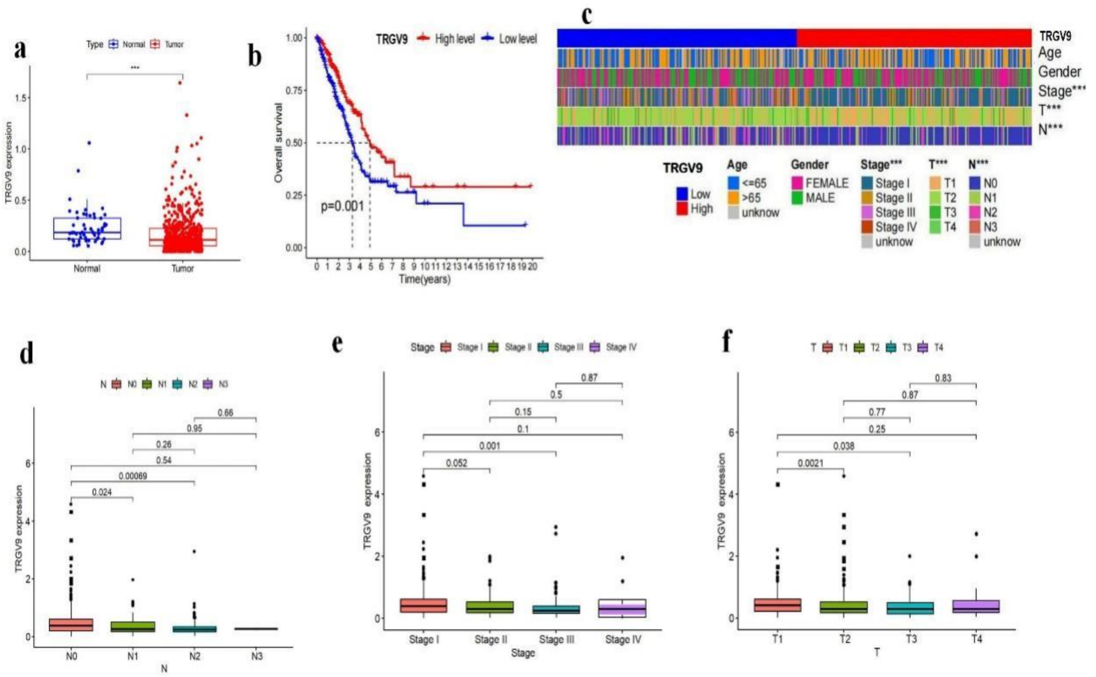

Fig.s5 Correlation analysis between differential TRGV9 expression and clinical features **a.** Analysis of TRGV9 expression differences between normal tissues and LUAD **b.** Survival analysis of TRGV9 in LUAD **c.** Heatmap illustrating the association of TRGV9 expression with age, gender, stage, T, and N of LUAD patients **d.** Correlation of TRGV9 expression with N staging **e.** Correlation of TRGV9 expression with stage **f.** Correlation of TRGV9 expression with T staging

Supplement Table 1 Impact of RNF125, CD8B, and TRGV9 on lung adenocarcinoma

exposure	outcome	method （heterogeneity）	P value	method （pleiotrop）
RNF125	lung adenocarcinoma	Inverse variance weighted	0.755703216	Egger_intercept
CD8B	lung adenocarcinoma	Inverse variance weighted	0.711380024	Egger_intercept
TRGV9	lung adenocarcinoma	Inverse variance weighted	0.571487335	Egger_intercept
